# Supplementary material for: Heterogeneity in Oct4 and Sox2 Targets Biases Cell Fate in 4-Cell Mouse Embryos
Source: Cell. 2016 Mar 24;165(1):61–74. doi: 10.1016/j.cell.2016.01.047 (PMC4819611; doi:10.1016/j.cell.2016.01.047)
Supplement: Document S1. Tables S1 and S2 [file mmc1.pdf]

Cell, Volume *165*

Supplemental Information

Heterogeneity in Oct4 and Sox2 Targets

Biases Cell Fate in 4-Cell Mouse Embryos

Mubeen Goolam, Antonio Scialdone, Sarah J.L. Graham, Iain C. Macaulay, Agnieszka Jedrusik, Anna Hupalowska, Thierry Voet, John C. Marioni, and Magdalena Zernicka-Goetz

Supplemental Tables:

Table S1. List of mRNA sequencing samples, related to Figure 1.

Sample Name	Stage	Division Pattern	Batch	QC
2cell_1_A, 2cell_1_B	2-cell		1	Passed
2cell_2_A, 2cell_2_B	2-cell		1	Passed
2cell_3_A, 2cell_3_B	2-cell		1	Passed
2cell_4_A, 2cell_4_B	2-cell		3	Passed
2cell_5_A, 2cell_5_B	2-cell		3	Passed
2cell_6_A, 2cell_6_B	2-cell		3	Passed
2cell_7_A, 2cell_7_B	2-cell		3	Passed
2cell_8_A, 2cell_8_B	2-cell		3	Passed
ME_4cell_1_A, ME_4cell_1_B, ME_4cell_1_C, ME_4cell_1_D	4-cell	ME	1	Passed
ME_4cell_2_A, ME_4cell_2_B, ME_4cell_2_C, ME_4cell_2_D	4-cell	ME	1	Passed
ME_4cell_3_A, ME_4cell_3_B, ME_4cell_3_C, ME_4cell_3_D	4-cell	ME	1	Passed
ME_4cell_4_A, ME_4cell_4_B, ME_4cell_4_C, ME_4cell_4_D	4-cell	ME	4	Passed
ME_4cell_5_A, ME_4cell_5_B, ME_4cell_5_C, ME_4cell_5_D	4-cell	ME	4	Passed
ME_4cell_6_A, ME_4cell_6_B, ME_4cell_6_C, ME_4cell_6_D	4-cell	ME	4	Passed
EM_4cell_1_A, EM_4cell_1_B, EM_4cell_1_C, EM_4cell_1_D	4-cell	EM	1	Passed
EM_4cell_2_A, EM_4cell_2_B, EM_4cell_2_C, EM_4cell_2_D	4-cell	EM	1	Passed
EM_4cell_3_A, EM_4cell_3_B, EM_4cell_3_C, EM_4cell_3_D	4-cell	EM	1	Passed
EM_4cell_4_A, EM_4cell_4_B, EM_4cell_4_C, EM_4cell_4_D	4-cell	EM	4	Passed
EM_4cell_5_A, EM_4cell_5_B, EM_4cell_5_C, EM_4cell_5_D	4-cell	EM	4	Passed
EM_4cell_6_A, EM_4cell_6_B, EM_4cell_6_C, EM_4cell_6_D	4-cell	EM	4	Passed
MM_4cell_1_A, MM_4cell_1_B, MM_4cell_1_C, MM_4cell_1_D	4-cell	MM	1	Passed

**Sample Name**	**Stage**	**Division Pattern**	**Batch**	**QC**
MM_4cell_2_A, MM_4cell_2_B, MM_4cell_2_C, MM_4cell_2_D	4-cell	MM	1	Passed
MM_4cell_3_A, MM_4cell_3_B, MM_4cell_3_C, MM_4cell_3_D	4-cell	MM	1	Passed
EE_4cell_1_A, EE_4cell_1_B, EE_4cell_1_C, EE_4cell_1_D	4-cell	EE	1	Passed
8cell_1_A, 8cell_1_B, 8cell_1_C, 8cell_1_D, 8cell_1_E, 8cell_1_F, 8cell_1_G, 8cell_1_H	8-cell		1	Passed
8cell_2_A, 8cell_2_B, 8cell_2_C, 8cell_2_D, 8cell_2_E, 8cell_2_F, 8cell_2_G, 8cell_2_H	8-cell		1	Passed
8cell_3_A, 8cell_3_B, 8cell_3_C, 8cell_3_D, 8cell_3_E, 8cell_3_F, 8cell_3_G, 8cell_3_H	8-cell		2	Passed
8cell_4_A, 8cell_4_B, 8cell_4_C, 8cell_4_D, 8cell_4_E, 8cell_4_F, 8cell_4_G, 8cell_4_H	8-cell		2	Passed
16cell_A	16-cell		1	Passed
16cell_B	16-cell		1	Passed
16cell_C	16-cell		1	Passed
16cell_D	16-cell		1	Passed
16cell_E	16-cell		1	Passed
16cell_F	16-cell		1	Passed
32cell_A	32-cell		1	Passed
32cell_B	32-cell		1	Passed
32cell_C	32-cell		1	Passed
32cell_D	32-cell		1	Passed
32cell_E	32-cell		1	Passed
32cell_F	32-cell		1	Failed

Table S2. List of genes that are heterogeneous at 2, 4 and 8 cell stages, related to Figure 1.

Ensembl Gene ID	Gene Name
ENSMUSG00000018733	Pex12
ENSMUSG00000024922	Ovol1
ENSMUSG00000035202	Lars2
ENSMUSG00000090744	Gm6871
ENSMUSG00000027079	Clp1
ENSMUSG00000036078	Sigmar1
ENSMUSG00000026049	Tex30
ENSMUSG00000041287	Sox15
ENSMUSG00000001270	Ckb
ENSMUSG00000043421	Hilpda
ENSMUSG00000033948	Zswim5
ENSMUSG00000004359	Spic
ENSMUSG00000021712	Trim23
ENSMUSG00000086324	Gm15564
ENSMUSG00000074733	5830428H23Rik
ENSMUSG00000061371	Zfp873
ENSMUSG00000067813	Xkr9
ENSMUSG00000025921	Rdh10
ENSMUSG00000101655	NA
ENSMUSG00000038496	Slc19a3
ENSMUSG00000028109	Hormad1
ENSMUSG00000064194	Zfp936
ENSMUSG00000101609	NA
ENSMUSG00000041961	Znrf3
ENSMUSG00000001525	Tubb5
ENSMUSG00000001555	Fkbp10
ENSMUSG00000064370	mt-Cytb
ENSMUSG00000051176	Zfp42
ENSMUSG00000033419	Snap91
ENSMUSG00000001558	Klhl10
ENSMUSG00000071302	2610044O15Rik8
ENSMUSG00000017550	Atad5
ENSMUSG00000037904	Ankrd9
ENSMUSG00000064351	mt-Co1
ENSMUSG00000040013	Fkbp6
ENSMUSG00000025838	Pramel6
ENSMUSG00000064337	mt-Rnr1
ENSMUSG00000025068	Gsto1
ENSMUSG00000017548	Suz12
ENSMUSG00000026675	Hsd17b7
ENSMUSG00000012640	Zfp715
ENSMUSG00000025764	Phf17

ENSMUSG00000030747	Dgat2
ENSMUSG00000029569	Tmem168
ENSMUSG00000027954	Efna1
ENSMUSG00000060935	AI597468
ENSMUSG00000019818	Cd164
