# Supplementary material for: Heterogeneity in Oct4 and Sox2 Targets Biases Cell Fate in 4-Cell Mouse Embryos
Source: Cell. 2016 Mar 24;165(1):61–74. doi: 10.1016/j.cell.2016.01.047 (PMC4819611; doi:10.1016/j.cell.2016.01.047)
Supplement: Document S2. Article plus Supplemental Information [file mmc3.pdf]

Cell

Article

Heterogeneity in Oct4 and Sox2 Targets Biases Cell Fate in 4-Cell Mouse Embryos

Graphical Abstract

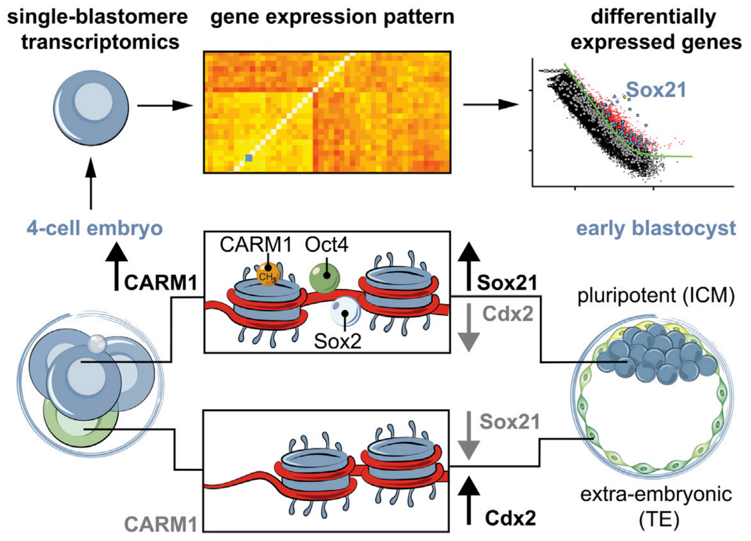

Highlights

- Oct4 and Sox2 targets such as Sox21 are highly heterogeneous in the 4-cell embryo
- Depleting Sox21 levels leads to upregulation of Cdx2 and extra-embryonic fate
- Sox21 expression responds to the histone H3R26 methyltransferase CARM1
- Heterogeneous gene expression initiates cell-fate decisions at the 4-cell stage

Authors

Mubeen Goolam, Antonio Scialdone, Sarah J.L. Graham, ..., Thierry Voet, John C. Marioni, Magdalena Zernicka-Goetz

Correspondence

marioni@ebi.ac.uk (J.C.M.), mz205@cam.ac.uk (M.Z.-G.)

In Brief

Heterogeneous gene expression in 4-cell stage in mouse embryos contributes to cell-fate decisions, with lower levels of Sox21 promoting extra-embryonic fate over pluripotency.

Accession Numbers

E-MTAB-3321

Goolam et al., 2016, Cell *165*, 61–74
March 24, 2016 ©2016 The Authors
http://dx.doi.org/10.1016/j.cell.2016.01.047

CellPress

Cell

Heterogeneity in Oct4 and Sox2 Targets Biases Cell Fate in 4-Cell Mouse Embryos

Mubeen Goolam,[1,6] Antonio Scialdone,[2,3,6] Sarah J.L. Graham,[1,6] Iain C. Macaulay,[3] Agnieszka Jedrusik,[1] Anna Hupalowska,[1] Thierry Voet,[3,4] John C. Marioni,[2,3,5,*] and Magdalena Zernicka-Goetz[1,*]
[1]Department of Physiology, Development & Neuroscience, University of Cambridge, Downing Street, Cambridge CB2 3EG, UK
[2]European Bioinformatics Institute, European Molecular Biology Laboratory (EMBL-EBI), Wellcome Genome Campus, Cambridge CB10 1SD, UK
[3]Wellcome Trust Sanger Institute, Wellcome Genome Campus, Cambridge CB10 1SA, UK
[4]Laboratory of Reproductive Genomics, Department of Human Genetics, KU Leuven, Herestraat 49, 3000 Leuven, Belgium
[5]Cancer Research UK-Cambridge Institute, University of Cambridge, Li Ka Shing Centre, Cambridge CB2 0RE, UK
[6]Co-first author
*Correspondence: marioni@ebi.ac.uk (J.C.M.), mz205@cam.ac.uk (M.Z.-G.)
http://dx.doi.org/10.1016/j.cell.2016.01.047
This is an open access article under the CC BY license (http://creativecommons.org/licenses/by/4.0/).

SUMMARY

The major and essential objective of pre-implantation development is to establish embryonic and extra-embryonic cell fates. To address when and how this fundamental process is initiated in mammals, we characterize transcriptomes of all individual cells throughout mouse pre-implantation development. This identifies targets of master pluripotency regulators Oct4 and Sox2 as being highly heterogeneously expressed between blastomeres of the 4-cell embryo, with Sox21 showing one of the most heterogeneous expression profiles. Live-cell tracking demonstrates that cells with decreased Sox21 yield more extra-embryonic than pluripotent progeny. Consistently, decreasing Sox21 results in premature upregulation of the differentiation regulator Cdx2, suggesting that Sox21 helps safeguard pluripotency. Furthermore, Sox21 is elevated following increased expression of the histone H3R26-methylase CARM1 and is lowered following CARM1 inhibition, indicating the importance of epigenetic regulation. Therefore, our results indicate that heterogeneous gene expression, as early as the 4-cell stage, initiates cell-fate decisions by modulating the balance of pluripotency and differentiation.

INTRODUCTION

When in mammalian development cells first start to differ from each other and whether these first differences play any role in cell-fate specification remain key open questions. In many model systems, initiation of cell-fate specification stems from heterogeneity between the blastomeres of the early embryo, but whether this might also be the case in mammals remains unknown. The first cell-fate specification in the mammalian embryo leads to the separation of embryonic and extra-embryonic lineages. The embryonic lineage is pluripotent and will give rise to the fetus, while the extra-embryonic lineages will differentiate into supportive structures critical for embryo implantation and fetal development, the placenta, and yolk sac (Takaoka and Hamada, 2012; Zernicka-Goetz et al., 2009). How and when these lineages start to separate in morphologically homogenous cells has been very difficult to dissect in mammals. Historically, cells of the early mouse embryo were considered identical in their ability to give rise to embryonic or extra-embryonic lineages, due to the regulative ability of the embryo to compensate for alterations in cell arrangement (Hillman et al., 1972; Tarkowski, 1959). However, more recent evidence has suggested that cells as early as the 4-cell stage become heterogeneous, exhibiting differences in developmental fate and potential (Bischoff et al., 2008; Piotrowska-Nitsche et al., 2005; Tabansky et al., 2013) and in the activity of specific cell-fate regulators (Burton et al., 2013; Plachta et al., 2011; Torres-Padilla et al., 2007). This heterogeneity indicates the possibility that the breaking of embryo symmetry starts earlier than expected, prior to differences in cell position and polarity evident from the 16-cell-stage onward (Fleming, 1987; Johnson and Ziomek, 1981). However, finding causal links between this early heterogeneity and later lineage divergence has proved extremely difficult because the key evidence—differences in gene expression patterns between individual cells that regulate cell fate—has, until now, been hard to identify due to technical limitations.

High-throughput single-cell transcriptomics offers an unbiased approach for understanding the extent, basis, and function of gene expression variation between seemingly identical cells. So far, the focus of single-cell studies in the mouse embryo has been on gene expression patterns that characterize particular developmental stages or lineages within the blastocyst or mono versus bi-allelic gene expression (Biase et al., 2014; Deng et al., 2014; Guo et al., 2010; Shi et al., 2015; Tang et al., 2011; Xue et al., 2013), rather than on investigating the functional consequences of heterogeneity within the same embryo for cell-fate specification. Here, using single-cell transcriptomics, we determined the extent of transcriptional heterogeneities between individual cells in pre-implantation embryos and identified that target genes of the pluripotency master regulators Oct4 and

Sox2 are highly heterogeneous at the 4-cell stage. We find that *Sox21*, a Sox2 target that also displays proximal binding of Oct4 and that regulates expression of the master regulator of differentiation Cdx2, is one of the most highly heterogeneous genes at this stage. We also find that decreasing expression of Sox21 in individual cells results in premature and increased expression of Cdx2 and decreased contribution to the pluripotent lineage. We identify CARM1, which methylates histone H3 at arginine 26 (H3R26), as an upstream regulator of Sox21 expression. These results indicate that heterogeneity in gene expression patterns biases cell fate in the mouse embryo as early as the 4-cell stage.

RESULTS

Temporal and Spatial Gene Expression Patterns in Single Cells during Pre-implantation Development

To assess gene expression patterns throughout the pre-implantation development of mouse embryos, we isolated individual cells from successive stages and assayed their transcriptomes using the Smart-Seq2 single-cell RNA-sequencing protocol (Picelli et al., 2014). Transcriptomes were determined for all blastomeres of 28 embryos isolated at the 2-cell (n = 8), 4-cell (n = 16), and 8-cell (n = 4) stages, and for individual cells at the 16-cell (n = 6) and 32-cell (n = 6) stages, corresponding to the morula and blastocyst, respectively (Figure 1A; Table S1). To assess the quality of the data we compared, across cells in each batch, three metrics: the fraction of reads mapped to endogenous RNA molecules, the number of genes with more than 10 reads per million, and the fraction of reads mapped to mitochondrial genes (Stegle et al., 2015). One 32-cell stage sample failed our quality check and was excluded from downstream analysis (Experimental Procedures; Figure S1).

Extrinsic "spike-in" RNA-molecules were added to each cell's lysate prior to cDNA conversion, amplification, and library preparation (Experimental Procedures). We applied principal component analysis (PCA) to the log-transformed normalized read counts from the spike-ins and, as expected, did not observe any structure in the data (Figure S2). When we applied PCA to the log-transformed normalized read counts of highly variable genes from all cells analyzed, the cells grouped by developmental stage (Figure 1B; Experimental Procedures), in accord with previous results (Deng et al., 2014; Tang et al., 2011; Xue et al., 2013).

To identify genes that are differentially expressed as development progresses, we analyzed temporal gene expression patterns throughout pre-implantation development. At a false discovery rate (FDR) cutoff of 0.05, 2,716 genes were differentially expressed between 2- and 4-cell, 3,222 between 4- and 8-cell, 2,030 between 8- and 16-cell, and 351 between 16- and 32-cell embryos. Consequently, our data suggest that the transition from the 4- to 8-cell stage is where most transcriptional changes occur. To quantitatively assess intra- and inter-embryo heterogeneity in gene expression, we focused on the stages where all individual cells could be collected from the same embryo (the 2-, 4-, and 8-cell stages). We computed the correlation of expression levels between all pairs of blastomeres within each complete embryo (intra-embryonic correlation) and compared this to the correlation of blastomeres between different embryos (inter-embryonic correlation). We found that intra-embryonic correlation was significantly higher than the inter-embryonic correlation for 2- and 4-cell embryos, while at the 8-cell stage no statistically significant difference could be identified (Figure 1C; Welch's t test, p values are 8.5×10^{-5}, 5.9×10^{-7} and 0.74 for the 2-, 4-, and 8-cell stage, respectively). Thus at the 2- and 4-cell stages inter-embryonic variability is higher than the transcriptional differences between cells of the same embryo. Conversely, at the 8-cell stage the differences between cells in the same embryo are comparable to those between cells from different embryos, suggesting a relatively higher degree of intra-embryonic heterogeneity. After correcting for inter-embryo differences in expression levels, we identified genes displaying significantly more intra-embryonic heterogeneity in expression than expected by chance (Experimental Procedures). These analyses revealed 659, 1,339, and 813 highly variable genes at 2-, 4-, and 8-cell embryos, respectively (Figures 1D and S3; Data S1). Of these variable genes, 47 showed heterogeneous expression at all three stages (Table S2), 82 are shared between the 2- and the 4-cell stage, 203 between the 4- and the 8-cell stage, and 31 between the 2- and the 8-cell stage (Figure 1D).

Previous studies have shown that embryo development can differ depending on the orientation and order of cell divisions to the 4-cell stage (Gardner, 2002; Piotrowska-Nitsche and Zernicka-Goetz, 2005), leading us to characterize gene expression in embryos with different division patterns (Figure S4). PCA analysis of 16 4-cell embryos revealed that embryos with distinct cell division patterns align approximately along the first principal component (Figure 1E; Figure S4). Thus, cell division orientation and order might be one of the factors contributing to gene expression patterns in the 4-cell embryo. However, here we focus on genes expressed highly heterogeneously between cells in all individual embryos, independent of their cell division pattern.

Sox21 mRNA Expression Is Highly Variable at the 4-Cell Stage and Correlates with the Expression of Pluripotency-Related Genes

We reasoned that highly heterogeneous genes in the 4-cell embryo were of particular interest as cells at this stage can display differential fate (Piotrowska-Nitsche et al., 2005; Bischoff et al., 2008; Plachta et al., 2011; Tabansky et al., 2013) and potential (Piotrowska-Nitsche et al., 2005; Morris et al., 2012). One of the most highly heterogeneous genes in all embryos analyzed at the 4-cell stage is the gene encoding the transcription factor Sox21 (Figure 2A), which is involved in regulating ES cell-fate downstream of Sox2 (Kuzmichev et al., 2012; Mallanna et al., 2010). Sox21 has not been previously studied in the early mouse embryo but is known to inhibit expression of the trophectoderm (TE) master gene Cdx2 in ES cells and is important for reprogramming (Kuzmichev et al., 2012). Furthermore, Sox21 expression is directly regulated by Sox2 (Chakravarthy et al., 2011; Kuzmichev et al., 2012; Mallanna et al., 2010), and its regulatory region is bound by Oct4 (Chakravarthy et al., 2011; Göke et al., 2011), which has heterogeneous nuclear-cytoplasmic kinetics at the 4-cell stage (Plachta et al., 2011). Together, this suggests that the heterogeneous Sox21 expression may itself be

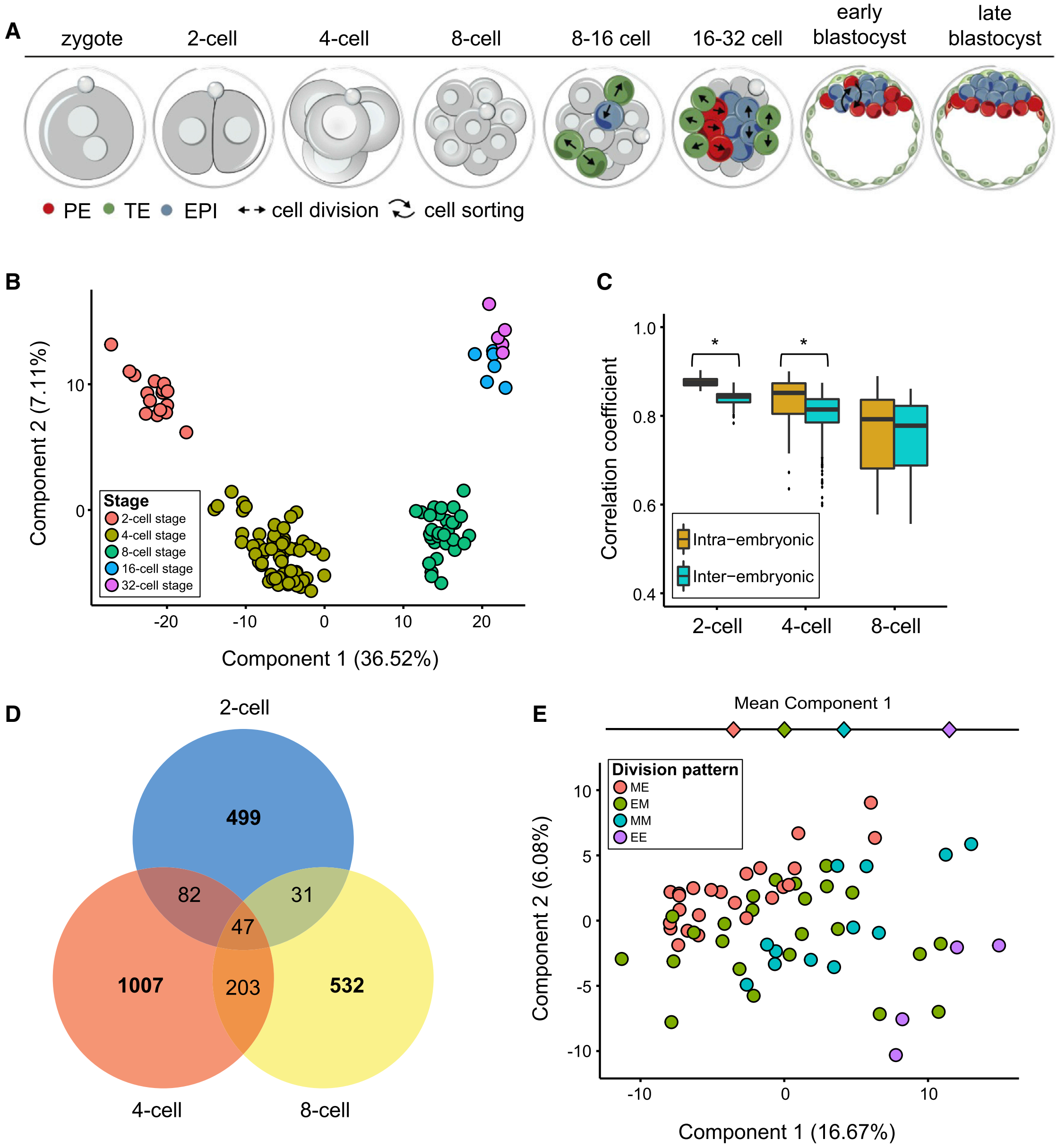

Figure 1. Cellular Heterogeneity in Overall Gene Expression Patterns

(A) Scheme of development from the fertilized egg (zygote) to the blastocyst stage with three lineages: epiblast (EPI; blue), primitive endoderm (PE, red), and trophectoderm (TE, green). The mechanism(s) initiating these cell-fate decisions remains unknown (reviewed in Zernicka-Goetz et al., 2009).

(B) Principal component analysis of the gene expression patterns of single cells at different stages. The percentage of variance explained by each principal component is indicated in parentheses.

(C) Spearman correlation coefficients of blastomeres within the same embryo (intra-embryonic) and in different embryos (inter-embryonic) at the 2-, 4-, and 8-cell stage. Asterisks indicate statistically significant differences (Welch's t test).

(D) Venn diagram with the number of genes having a significantly high degree of heterogeneous expression at 2-, 4-, and 8-cell stages.

(E) Principal component analysis of the transcriptomes of cells from embryos at the 4-cell stage with different division patterns: ME, six embryos; EM, six embryos; MM, three embryos; EE, one embryo. M, meridional; E, equatorial cell division.

See also Figures S1, S2, S3, and S4, Tables S1 and S2, and Data S1.

regulated by heterogeneous Oct4 and/or Sox2 activity in the embryo. To explore whether Oct4 and/or Sox2 could drive heterogeneity in gene expression, we next determined whether their target genes are overrepresented in the "highly heterogeneous" group of genes, and whether their expression correlates with that of *Sox21*, which would be expected if they are regulated by differential Oct4 and/or Sox2 activity in a cell-specific manner.

Cell

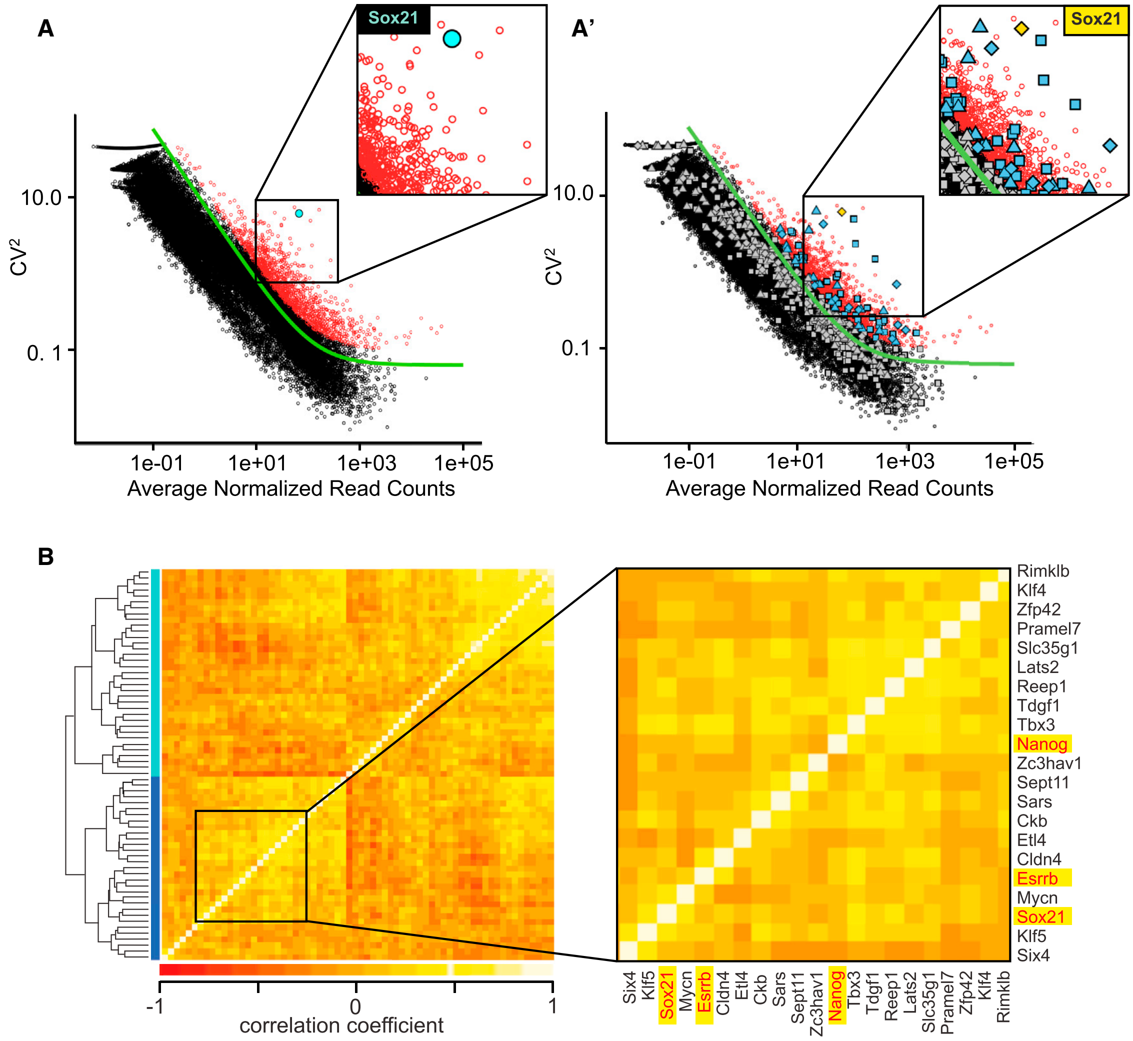

Figure 2. Heterogeneous Expression of Oct4 and Sox2 Target Genes at the 4-Cell Stage

(A) To find highly variable genes, we used the parameterization $CV^2 = a_1/\mu + \alpha_0$ to fit the relationship between the square of the coefficient of variation, CV^2, and the average expression level μ (green continuous curve; see Experimental Procedures for further details). All highly variable genes (with an adjusted p value < 0.1) marked by red circles. Sox21, cyan circle.

(A′) The same information from (A) but with Oct4 and/or Sox2 targets indicated (diamonds mark shared Sox2 and Oct4 targets, squares mark Oct4 targets, and triangles mark Sox2 targets). Gray symbols mark Oct4 and/or Sox2 targets that are not variable and blue symbols mark Oct4 and/or Sox2 targets that are highly variable at the 4-cell stage. Sox21 is marked in gold.

(B) Heatmap showing the Spearman correlation coefficient among highly variable Oct4 and/or Sox2 targets. A dynamic tree cut algorithm identified two clusters of genes showing similar patterns of correlations (colored side bars; Experimental Procedures).

See also Figure S5.

We applied Fisher's exact test to a list of Oct4/Sox2 downstream target genes (Experimental Procedures) and found that they are indeed overrepresented in the set of highly variable genes at the 4-cell stage (p value 3×10^{-10}; Figure 2A′). Furthermore, we found that a subset of highly variable Oct4/Sox2 target genes are correlated with Sox21 (Figures 2B and S5), suggesting coordinated expression of this module of genes at this stage. Interestingly, this module includes known pluripotency-related genes, such as *Nanog* and *Esrrb*, suggesting that *Sox21* expression might be associated with a "more pluripotent" transcriptional state.

When we examined the mRNA expression of *Sox21* through time, we observed a peak at the 4-cell stage followed by downregulation from the 8-cell stage onward (Figure 3A). By contrast, the expression of *Esrrb* and *Nanog* increases at the 8- and 16-cell stages, respectively (Figure 3A). The expression pattern of *Sox21* mRNA was consistently highly variable, with a large difference in expression between the highest and lowest expressing cells across all 4-cell embryos examined (Figure 3B; n = 64 cells, 16 embryos). Interestingly, in all cases there was at least one cell in each embryo with very low or no *Sox21* mRNA (Figure 3C). This cellular heterogeneity in Sox21 expression was confirmed at the

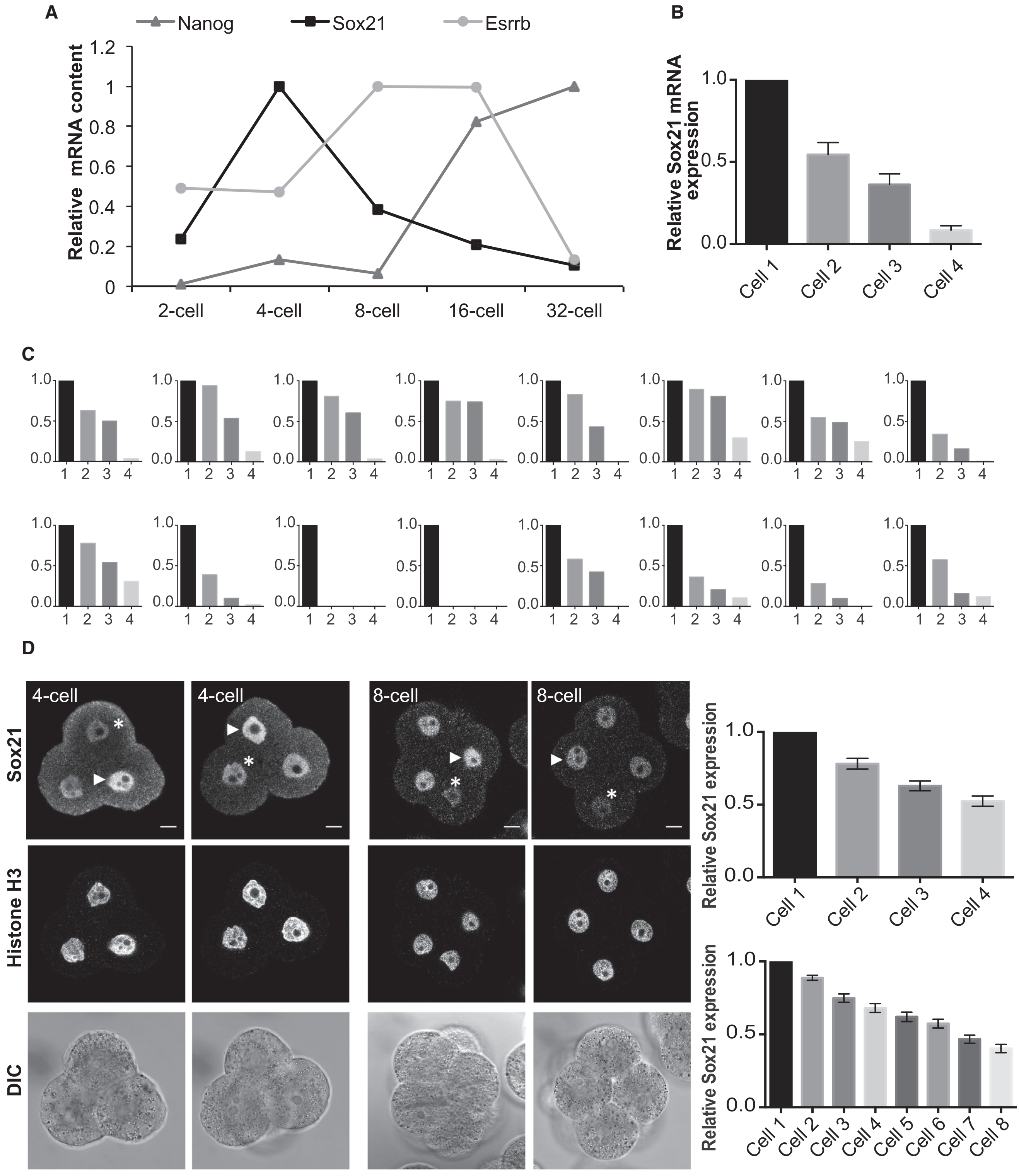

Figure 3. Sox21 mRNA and Protein Expression Is Heterogeneous and Peaks at the 4-Cell Stage

(A) Relative mRNA expression of Sox21, Nanog, and Esrrb.

(B) Average relative Sox21 mRNA levels in 4-cell embryos.

(legend continued on next page)

protein level at both the late 4- and early 8-cell stages (Figure 3D; n = 25 4-cell embryos and n = 23 8-cell embryos). These results, together with the role of Sox21 in regulating ES cell fate (Kuzmichev et al., 2012), led us to investigate whether heterogeneous Sox21 expression might have functional consequences for cell-fate specification in the embryo.

Live-Cell Tracking Demonstrates Cells with Decreased Sox21 Expression Contribute More Extra-Embryonic than Pluripotent Progeny

To determine whether heterogeneity in Sox21 expression influences cell fate, we decreased Sox21 expression using a combination of three *Sox21* small interfering RNAs (siRNAs), which we confirmed reduce Sox21 protein to undetectable levels (Figure 4A). To generate Sox21 heterogeneity, we injected these siRNAs into single blastomeres at the late 2-cell stage alongside mRNA for GFP as a lineage marker to follow a cell's developmental fate (Figure 4B). We allowed the embryos to develop for 72 hr, until the late blastocyst stage, and scored the lineage contribution of each cell using molecular markers for each lineage and cell position within the embryo (Figure 4C). Cells with decreased Sox21 expression were significantly more likely to initiate differentiation and contribute to the extra-embryonic TE lineage ($p < 0.001$) than cells injected with control siRNA, and correspondingly significantly less likely to contribute to pluripotent inner cell mass (ICM) lineages ($p < 0.01$, Figure 4D, n = 75 and n = 38 embryos for *Sox21* siRNA and control, respectively). When we injected each *Sox21* siRNA individually, the same phenotype was observed (Figure S6), indicating that the phenotype is specific to decreased Sox21 expression and not due to off-target effects.

Segregation of the TE and ICM occurs primarily as a result of asymmetric cell divisions at the 8–16 and 16–32 cell transitions, which contribute one daughter cell to the TE and one to the ICM (Fleming, 1987; Johnson and Ziomek, 1981; Morris et al., 2010; Watanabe et al., 2014). Inside cells can also be generated by apical constriction or cell engulfment (McDole et al., 2011; Morris et al., 2012; Parfitt and Zernicka-Goetz, 2010; Samarage et al., 2015; Yamanaka et al., 2010). To determine the cellular mechanism by which cells with decreased Sox21 expression contribute to the TE, we injected one blastomere of 2-cell embryos with three *Sox21* siRNAs, or control siRNA, together with mRNA for a GFP-tagged membrane-bound protein (Gap43-GFP) as a marker, and filmed development of the embryos by time-lapse microscopy (Figures 4E and 4F; n = 18 embryos, 288 cells for control siRNA and n = 16 embryos, 256 cells for *Sox21* siRNA). Live-cell tracking of all Gap43-GFP-expressing cells revealed that, by the early blastocyst (32-cell) stage, control siRNA blastomeres had generated an average of 10.9 outside cells and 5.1 inside cells (Figure 4G), with an average of 4.41 asymmetric divisions and 0.69 cells internalized by cell engulfment (Figures 4H and 4I). In contrast, the *Sox21* siRNA blastomeres had generated an average of 13.7 outside cells and only 2.3 inside cells, with significantly fewer asymmetric divisions (an average of 2.3) by the same stage ($p < 0.001$; Figures 4G, 4H, and 4J). Therefore, cells with lower Sox21 expression undertake fewer asymmetric cell divisions and contribute more extra-embryonic rather than pluripotent progeny.

Decreased Expression of Sox21 Results in Premature and Increased Expression of Cdx2

Correct differentiation into extra-embryonic TE requires the transcription factor Cdx2 (Jedrusik et al., 2008; Strumpf et al., 2005). As Sox21 has been shown to bind to an enhancer of *Cdx2* and repress its expression in ES cells (Kuzmichev et al., 2012), we next investigated whether Sox21 might also regulate *Cdx2* in the embryo. To this end, we first decreased expression of Sox21 at the zygote stage, by injecting a mixture of three siRNAs as before, and examined the developmental timing and level of *Cdx2* expression. Decreased expression of Sox21 resulted in a significant increase in *Cdx2* mRNA expression at the 8-cell stage, compared to control siRNA embryos (Figures 5A and 5B; $p < 0.05$; n = 85 and n = 75 embryos for Sox21 siRNA and control, respectively, three biological replicates). When we repeated the experiment by downregulating Sox21 in the zygote but now examined Cdx2 protein at the early 8-cell stage, when Cdx2 protein is normally not yet detectable, we found that all embryos in which Sox21 expression was decreased already expressed Cdx2 protein (Figures 5C and 5D; n = 96 cells [84 Cdx2-positive cells, 87.5%], 12 embryos for the experimental group and n = 88 cells [11 Cdx2-positive cells, 12.5%], 11 embryos for the control group).

Finally, to test the functional consequences of heterogeneous Sox21 expression on Cdx2 expression, we decreased Sox21 in just half of the embryo, from the 2-cell stage onward, using Gap43-RFP as a lineage marker (Figures 5E and 5F). This resulted in a significant upregulation of Cdx2 in the half of the embryo in which cells had decreased Sox21 expression (Figure 5G; $p < 0.001$; n = 104 cells, 13 embryos and n = 88 cells, 11 embryos for the experimental and control groups, respectively). Together, these results suggest that cells with lower Sox21 levels are the first to upregulate Cdx2 (mRNA and protein) and therefore are first to initiate development into the extra-embryonic TE lineage.

Sox21 Expression Is Regulated by CARM1 Activity

Since our results indicate that heterogeneity in Sox21 expression could bias cell fate in the early embryo, we sought to determine the upstream regulators of this heterogeneity. One factor we previously found to be heterogeneous at the 4-cell stage is histone H3R26 methylation mediated by CARM1, which itself is heterogeneously active at the 4-cell stage (Torres-Padilla et al., 2007). Furthermore, elevated CARM1 expression leads to increased histone H3R26me and contribution to the ICM (Torres-Padilla et al., 2007; Wu et al., 2009). To determine

(C) Relative Sox21 mRNA expression in all individual 4-cell embryos.
(D) Immunofluorescence of Sox21 in 4-cell (n = 25) and 8-cell (n = 23) embryos.
Fluorescence quantified and normalized to the nucleus with the strongest staining per individual 4-cell or 8-cell embryo. Arrowheads indicate highest expressing cell. Asterisks indicated lowest expressing cells. Error bars represent SEM. Scale bars, 10 μm.

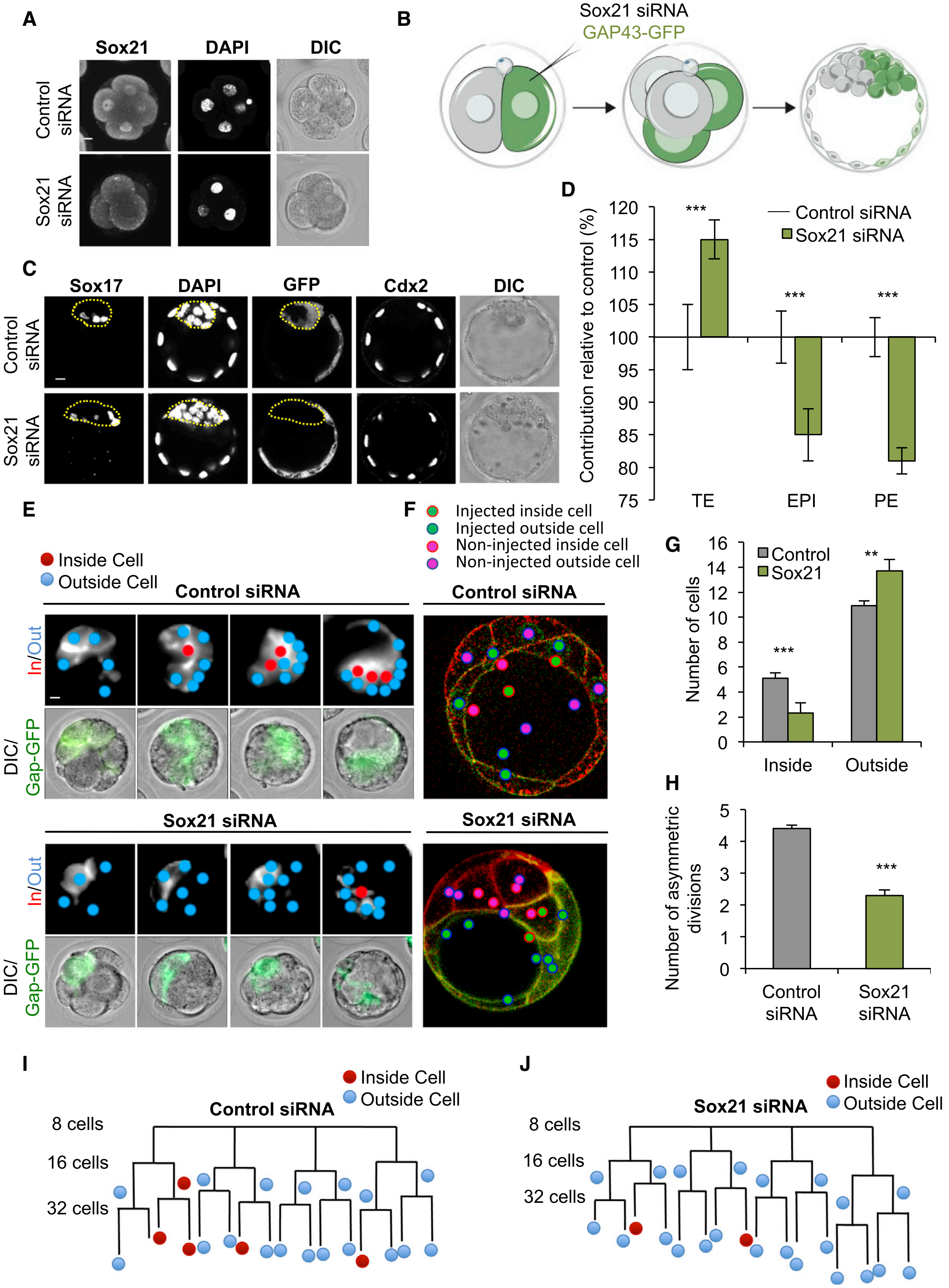

Figure 4. Decreasing Sox21 Expression Leads Cells to an Extra-Embryonic Fate

(A) Verification of *Sox21* siRNA efficiency at the 4-cell stage (Control siRNA n = 20, *Sox21* siRNA n = 21). The nuclear immunofluorescence signal is lost in embryos injected with *Sox21* siRNA.

(B) Scheme of clonal *Sox21* siRNA. One blastomere of 2-cell stage embryos injected with *Sox21* siRNA or control siRNA, and GFP mRNA. Embryos cultured to the late blastocyst stage and the contribution of the injected cells' progeny to each lineage analyzed.

(legend continued on next page)

whether altering CARM1 activity affects the expression of Sox21, we first treated embryos with a CARM1-specific inhibitor from the 2-cell stage. To test its effectiveness, we determined the levels of histone H3R26me after 10 hr of treatment and observed it to be reduced to an undetectable level (Figure 6A). Inhibition of CARM1 resulted in a complete loss of Sox21 expression at the 4-cell stage (Figure 6B, n = 21 control; n = 15 CARM1 inhibition). To downregulate CARM1 through an alternative mechanism, we injected *Carm1* siRNA into zygotes and cultured embryos until the blastocyst stage. Decreasing CARM1 expression resulted in a significant reduction in the number of pluripotent epiblast cells with a concurrent increase in the number of primitive endoderm cells and no effect on the TE lineage (Figures 6C and 6D $p < 0.001$; n = 12 control; n = 8 *Carm1* siRNA). To overexpress CARM1, we injected synthetic mRNA for CARM1 into zygotes. CARM1 upregulation led to a significant upregulation of *Sox21* mRNA expression at the 8-stage, as well as increased expression of other pluripotency genes, including *Sox2* and *Nanog* (Figure 6E; $p < 0.01$ and $p < 0.05$; n = 75 embryos and n = 66 embryos for CARM1 mRNA and control, respectively, three biological replicates). These results indicate that CARM1 activity regulates expression of a number of pluripotency regulators including Sox21 in the 4-cell embryo.

DISCUSSION

When heterogeneity in gene expression patterns first arises during the development of the mammalian embryo and how this might influence cell fate have remained, thus far, unknown. Approaching these questions has been difficult because of the inherent developmental flexibility of the embryo and because of previous technical limitations that prevented accurate quantification of gene expression levels in individual cells as development progresses. Although it has previously been thought that cells are homogenous until generation of inside and outside cells, more recent studies have opened a new possibility that cells at the 4-cell stage can already exhibit differences in cell fate (Piotrowska-Nitsche et al., 2005; Tabansky et al., 2013) and developmental potential (Morris et al., 2012; Piotrowska-Nitsche et al., 2005). This led to the suggestion that the differential activity of epigenetic regulators such as CARM1 (Torres-Padilla et al., 2007) or PRDM14 (Burton et al., 2013), or the differential behavior of transcription factors such as Oct4 (Plachta et al., 2011), at the 4-cell stage could be linked to differential cell fate and potential. Here, we sought to address this by first investigating and directly comparing global differences in gene expression between individual cells within the embryo during the first days of development. Focusing on the transcriptomes of each and every cell in 16 4-cell stage embryos uncovered consistent patterns of highly heterogeneous gene expression, with the most heterogeneously expressed genes being targets of Sox2 and Oct4, among which is the gene for the transcription factor Sox21.

The role of Sox21 in the early embryo has not been previously investigated, but studies in ES cells indicated its involvement in stem cell differentiation, the inhibition of Cdx2 expression, and cellular reprogramming (Mallanna et al., 2010, Kuzmichev et al., 2012). The regulatory regions of the *Sox21* gene are targets of Oct4 binding, and its expression is regulated by Sox2 (Chakravarthy et al., 2011; Göke et al., 2011; Kuzmichev et al., 2012; Mallanna et al., 2010). Among other target genes of Oct4 and/or Sox2 showing similar heterogeneity of expression, we identified those for pluripotency transcription factors such as Nanog and Esrrb; however, their expression peaked later than that of *Sox21*, suggesting that Sox21 could play a role very early in development. Indeed, our finding that expression of Sox21 represses premature differentiation in the embryo accords with the finding that Sox21 is essential for reprogramming in vitro (Kuzmichev et al., 2012). However, *Sox21* mutant mice are viable (Kiso et al., 2009). One possible explanation of this paradox could be that Sox21 is part of a functionally redundant mechanism typical of crucial biological processes and of importance in the regulative development of mammalian embryos. Heterogeneous expression of *Sox21*, along with other co-regulated genes, could also set up a competitive advantage between blastomeres during early development without being essential. Thus, while embryos may not have an absolute requirement for Sox21, heterogeneous Sox21 expression levels could lead to biased cell-fate decisions. We tested this possibility by setting up clonal differences in Sox21 levels experimentally. This revealed that clones of cells with decreased Sox21 expression contribute more differentiated TE rather than pluripotent progeny via a decreased frequency of asymmetric cell divisions. It has been previously shown that the frequency of symmetric to asymmetric cell divisions can be altered by the expression of the TE-specific transcription factor Cdx2 (Jedrusik et al., 2008). In agreement, we find that decreased Sox21 expression leads to elevated expression of Cdx2 at the 8-cell stage. This can be explained by noting that Sox21 binds to the *Cdx2* enhancer in stem cells to negatively regulate its expression (Kuzmichev et al., 2012). In addition, depletion of Sox21 in the whole embryo leads to earlier expression of Cdx2. Thus, in the case of uniform Sox21 depletion, there are no heterogeneities present and thus no

(C) Confocal images of control (n = 38) and *Sox21* (n = 75) siRNA embryos. Sox17 (primitive endoderm, PE) and Cdx2 (TE) used as lineage markers. Dotted lines mark the ICM.

(D) Contribution of *Sox21* siRNA cells to TE, PE, and EPI, relative to control siRNA cells.

(E) Time-lapse study following *Sox21* siRNA (n = 16 embryos, 256 cells) or control siRNA (n = 18 embryos, 288 cells). Gap43-GFP expression indicates blastomeres injected with either control or *Sox21* siRNA. Fluorescence images are overlaid with cell-tracking spheres.

(F) Example slice through embryos showing inside and outside cell fate of either control or *Sox21* siRNA blastomeres.

(G) Number of inside and outside cells contributed from injected blastomeres at the 32-cell stage.

(H) Number of asymmetric divisions injected blastomeres underwent from the 8- to 32-cell stage.

(I and J) Lineage trees from two representative embryos. All injected cells were traced to the 32-cell stage. A cell was defined as occupying an inside position when enclosed from the outside environment by neighboring cells. Inside or outside cell fate indicated.

Error bars represent SEM. Wilcoxon rank-sum test was used to test significance $^{**}p < 0.01$, $^{***}p < 0.001$. Scale bars represent 10 μm. See also Figure S6.

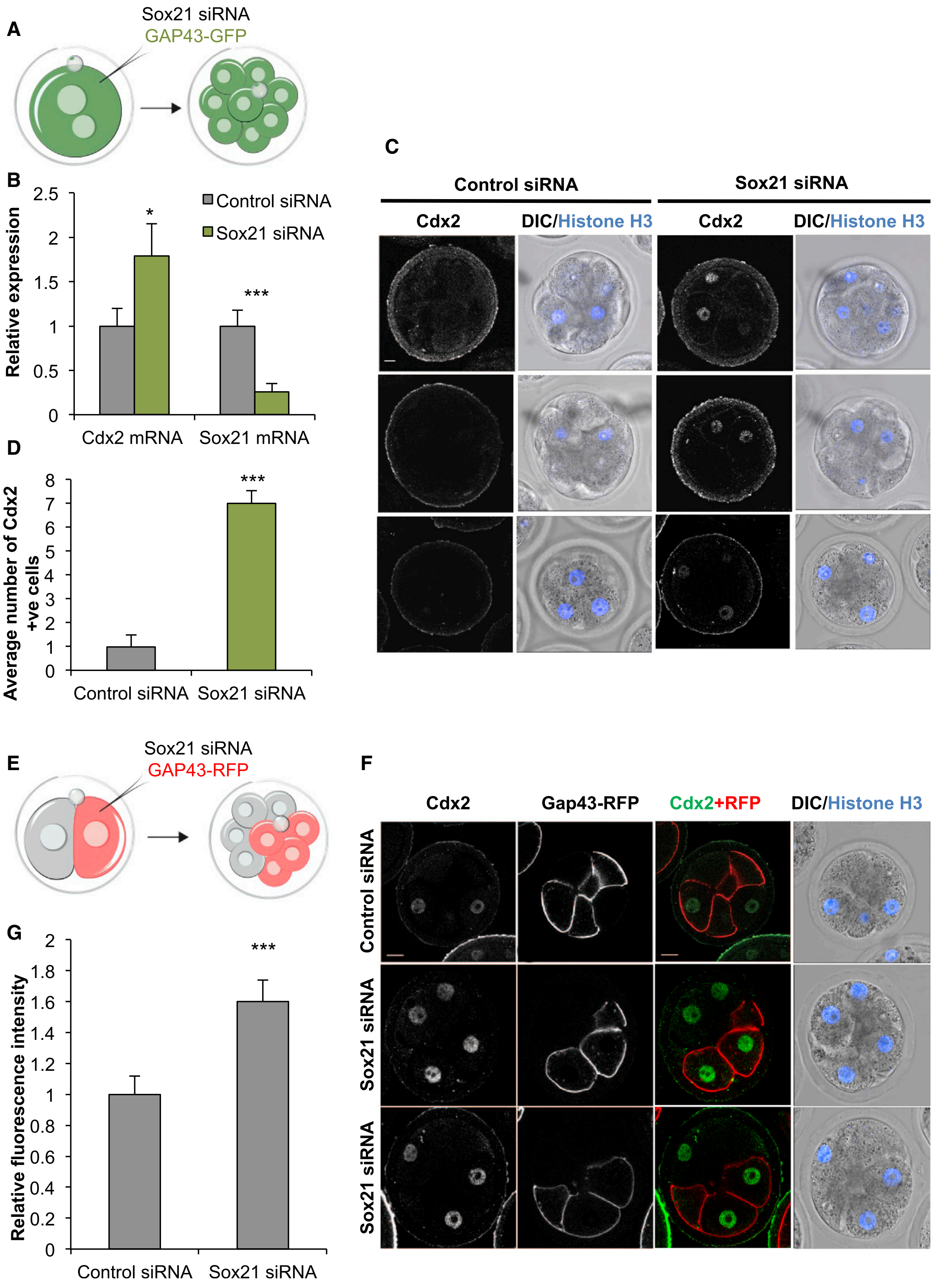

Figure 5. Sox21 Depletion Prematurely Upregulates Cdx2 Expression

(A) Scheme of *Sox21* siRNA experiment. Zygotes injected with *Sox21* siRNA, or control siRNA, and isolated at the 8-cell stage for immunostaining or qRT-PCR.

(B) qRT-PCR of embryos injected with either control siRNA (n = 75 embryos, three biological replicates) or *Sox21* siRNA (n = 85 embryos, three biological replicates) comparing mRNA expression of Cdx2 and Sox21 at the late 8-cell stage. Student's t test was used to test significance *$p < 0.05$, ***$p < 0.001$.

(C) Confocal images of Cdx2 and Histone H3 expression in control (n = 12) and *Sox21* (n = 12) siRNA embryos at the early 8-cell stage.

(legend continued on next page)

bias in cell fate. These results suggest that Sox21 expression in the 4-cell embryo might help to safeguard against the premature initiation of differentiation.

How might the heterogeneous expression of Sox21 first arise? It is possible that the Sox21 heterogeneity we identify here is generated by random transcriptional differences; however, we wished to investigate whether this differential expression could be regulated by factors previously found to be heterogeneous at the 4-cell stage. Our best candidate for the heterogeneous regulation of Sox21 was methylation of histone H3R26 because we have previously found that CARM1, which mediates this modification, regulates the expression of pluripotency genes and is differentially active in the 4-cell embryo (Torres-Padilla et al., 2007). We find here that inhibiting CARM1 activity results in a loss of Sox21 expression in 4-cell embryos and, in contrast, its upregulation causes an increase in expression of *Sox21* and the pluripotency genes *Sox2* and *Nanog* at the 8-cell stage. Therefore, differential activity of CARM1 in the 4-cell embryo is likely to be responsible for the differential expression of Sox21. We also show that by decreasing CARM1 expression we significantly reduce the number of pluripotent epiblast cells, indicating the critical role of CARM1 for maintaining the pluripotent state in the ICM. How CARM1 becomes heterogeneous remains an open question. The work presented here shows that CARM1 overexpression does not affect *Oct4* mRNA expression, suggesting that the regulation of *Sox21* by CARM1 is not achieved through transcriptional regulation of *Oct4*. This, however, does not exclude the possibility that CARM1 is indirectly affecting Oct4 or Sox2 activity. It can be hypothesized that increased levels of CARM1, resulting in increased histone H3R26me, could influence Oct4 and/or Sox2 DNA binding and lead to the upregulation of Oct4 and/or Sox2 target genes (Figure 7). Indeed, Oct4 expression is not heterogeneous at the 4-cell stage and its different DNA binding dynamics in individual cells might be due to differential accessibility to target genes (Plachta et al., 2011).

In conclusion, we suggest that highly heterogeneous expression of several Oct4 and/or Sox2 targets within the 4-cell embryo, regulated by differential activity of CARM1, creates heterogeneities that bias cell fate toward either an embryonic (pluripotent) or extra-embryonic (differentiating) fate. We further suggest that the highly heterogeneous expression of Sox21 is one of a number of mechanisms available to the embryo to direct cell fate. Our findings that several Oct4 and Sox2 target genes are overrepresented in the subset of highly variable genes at the 4-cell stage and that Sox21 is co-regulated with other transcription factors, such as Nanog and Esrrb, supports this hypothesis. Thus, if a single source of heterogeneity were to be removed, the embryo could compensate for this loss. In summary, our results indicate that heterogeneous gene expression as early as the 4-cell stage initiates cell-fate decisions.

EXPERIMENTAL PROCEDURES

Collection of Mouse Embryos and Cells

All animal experiments were performed in compliance with Home Office regulations. Embryos were recovered from superovulated F1 (C57Bl6xCBA) females into M2 media supplemented with 4% BSA, as described previously (Piotrowska et al., 2001). Individual cells were collected at late 2-cell (48 hr after hCG), late 4-cell (10 hr after the first 2-cell blastomere divided), late 8-cell (10 hr after the first 4-cell blastomere divided), 16-cell (78 hr after hCG), and 32-cell (86 hr after hCG) stages. Embryos for cell isolation at the 2-, 4,- and 8-cell stages were collected at the 2-cell stage and cultured in KSOM media (Millipore) at 37°C with 5% CO_2. Embryos for the 16- and 32-cell blastomeres were collected directly at the respective stages. The zona pellucida was removed using Tyrode's solution (Sigma). Zona-free embryos were incubated for 5 min (for the 2-, 4-, and 8-cell stages) or 20 min (for the 16- and 32-cell stages) in Ca^{2+} and Mg^{2+} free M2 before disaggregation by careful pipetting. Each embryo was processed individually. Single blastomeres were placed into individual tubes containing 2.3 μl of 0.2% Triton X-100 (Sigma) supplemented with 1 U/μl RNAsIN (Ambion).

Scoring 2- to 4-Cell Division Pattern

At the late 2-cell stage, one cell was injected with rhodamine-dextran (Invitrogen), as described previously (Bischoff et al., 2008). Embryos were observed at 20 min intervals to categorize the division order and orientation, meridional (M) or equatorial (E), relative to the position of the second polar body. 4-cell embryos were sorted into ME, EM, EE, and MM groups. Embryos with absent or unattached polar body were discarded. Following disaggregation, the cells were inspected under an epifluorescent microscope and classified as originating from the first- or second-dividing 2-cell blastomere based on the rhodamine fluorescence.

RNA Sequencing and Mapping of Reads

mRNA from the single cells was amplified using the SMARTSeq2 protocol (Picelli et al., 2014), with the additional inclusion of ERCC spike-in control (1 μl of 1:250,000 [batches 1 and 2] or a 1:1,000,000 [batch 3 and batch 4] dilution of mix 1 [Ambion] per cell). Multiplex sequencing libraries were generated from amplified cDNA using Nextera XT (Illumina) and sequenced on a HiSeq 2500 running in rapid mode. Paired-end reads were mapped simultaneously to the *M. musculus* genome (Ensembl v.38.77) and the ERCC sequences using GSNAP (v.2014-10-07) with default parameters. Htseq-count (Anders et al., 2015) was used to count the number of reads mapped to each gene (default options).

Quality Assessment of Cells

To assess the quality of the data, three metrics were used: the fraction of mapped reads, the number of genes with more than 10 reads per million (RPM), and the fraction of reads mapped to mitochondrial genes. Figures S1A–S1C show these metrics as functions of the total number of reads for each cell. When PCA was carried out using these three quantities, cells that perform worse than the average according to all three criteria appear as outliers (Figure S1D). One clear outlier was found (black arrow in all four panels) and was excluded from downstream analyses (sample name "32cell_F", see Table S1 for a list of samples).

Normalization of Read Counts and Analysis of Spike-ins

The data were divided into two groups according to whether they were mapped to ERCC spike-ins or to endogenous genes. For each of these groups a

(D) Quantification of the number of Cdx2 positive cells from (C). Student's t test was used to test significance ***$p < 0.001$.

(E) Scheme of *Sox21* siRNA experiment. One blastomere of 2-cell stage embryos was injected with *Sox21* siRNA or control siRNA and Gap43-RFP mRNA and the 8-cell embryos isolated.

(F) Confocal images of Cdx2 and Histone H3 expression in late 8-cell stage embryos after injection of one blastomere at the 2-cell stage with either control siRNA (n = 11) or *Sox21* siRNA (n = 13). Gap43-RFP expression on the membrane identifies injected cells.

(G) Quantification of relative fluorescence intensity of Cdx2 staining from (F).

Wilcoxon rank-sum test was used to test significance ***$p < 0.001$. Scale bars, 10 μm. Error bars represent SEM.

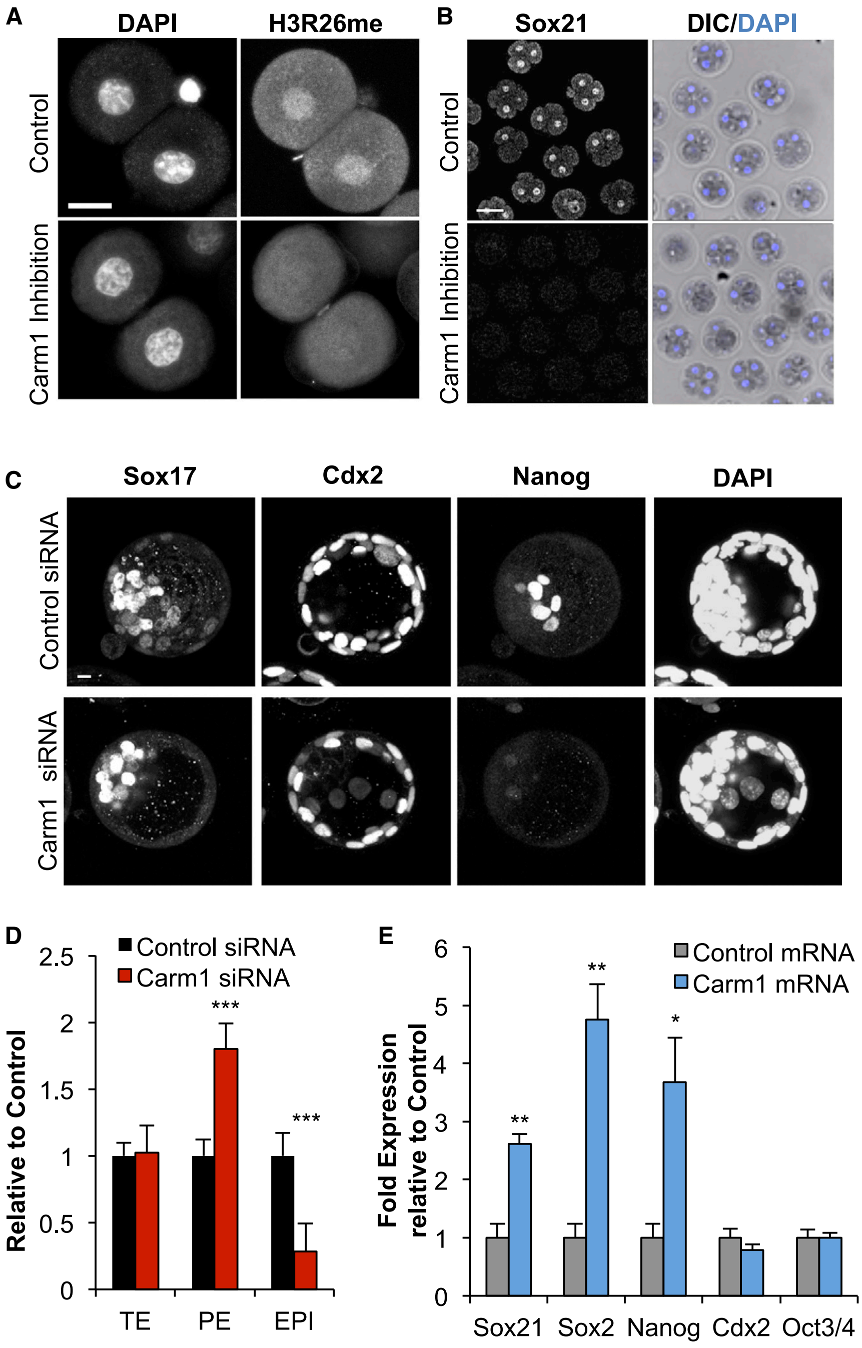

Figure 6. Sox21 Expression Is Regulated by CARM1 Activity

(A) Verification of CARM1 inhibition by staining for the presence of H3R26me (control n = 21, CARM1 inhibition n = 15). Scale bars, 20 μm.

(B) Confocal images of Sox21 expression in 4-cell embryos after being treated from the 2-cell stage with either DMSO control (n = 27) or CARM1 inhibitor (n = 24). Scale bar, 50 μm.

(C) Confocal images of control (n = 12) and *Carm1* siRNA (n = 8) embryos. Sox17 (PE), Cdx2 (TE), and Nanog (EPI) used as lineage markers. Scale bars, 10 μm.

(D) Overall number of TE, PE, and EPI cells present in *Carm1* siRNA-injected embryos, relative to control siRNA cells. Student's t test was used to test significance ***p < 0.001.

(E) qRT-PCR of embryos injected with either control mRNA (n = 66 embryos, three biological replicates) or CARM1 mRNA (n = 75 embryos, three biological replicates).

Embryos injected at the zygote stage and isolated for qRT-PCR at the late 8-cell stage Student's t test was used to test significance *p < 0.05, **p < 0.01. Error bars represent SEM.

set of size factors (Love et al., 2014) was calculated and used to normalize the raw read counts. While the size factors calculated with ERCC spike-ins account for sequencing depth, the size factors calculated on endogenous genes also normalize for the amount of RNA obtained from each cell (Brennecke et al., 2013), which is highly correlated with cell cycle stage (Buettner et al., 2015).

Differential Expression Analysis

First, genes with an average expression level of less than 50 normalized read counts across the two groups of cells compared were removed. The Bioconductor package DESeq2 (Love et al., 2014) was then used to find sets of differentially expressed genes. An FDR of 0.05 was used as a threshold for significance. To avoid confounding effects due to embryos being collected in different batches, when comparing blastomeres from different stages, we used only embryos at 2-, 4-, 8-, 16-, and 32-cell stages from the same batch (batch 1, see Table S1 for a list of samples).

Highly Variable Genes

To identify highly variable genes across all cells collected, we applied the method described in Brennecke et al. (2013) by fitting the squared coefficient of variation CV^2 as a function of the mean normalized counts μ with the parameterization $CV^2 = a_1/\mu + \alpha_0$. To minimize the skewing effect due to lowly expressed genes, only genes with a mean normalized count greater than 10 were used. Genes with an adjusted p value (Benjamini-Hochberg method) less than 0.1 were considered as significantly highly variable.

Adjusting for Batch Effects

The technical variation between samples in different batches was removed using the ComBat function in the R package "sva" with default options (Johnson et al., 2007). A $\log_{10}$ transformation was first applied to normalized read counts (by summing 1 to avoid infinities) and lowly expressed genes (less than 10 normalized read counts on average) were removed. ComBat was used to adjust for batch effect with the known batch covariate, controlling for sample stage and cell division pattern. All expression values that were 0 or negative after batch effect removal were set to 0. These batch-adjusted expression values were used to perform PCA on the highly variable genes. The results do not significantly change if different criteria are adopted to select genes (e.g., union of 1,000 or 5,000 most highly expressed genes, 3,000 genes with highest average expression).

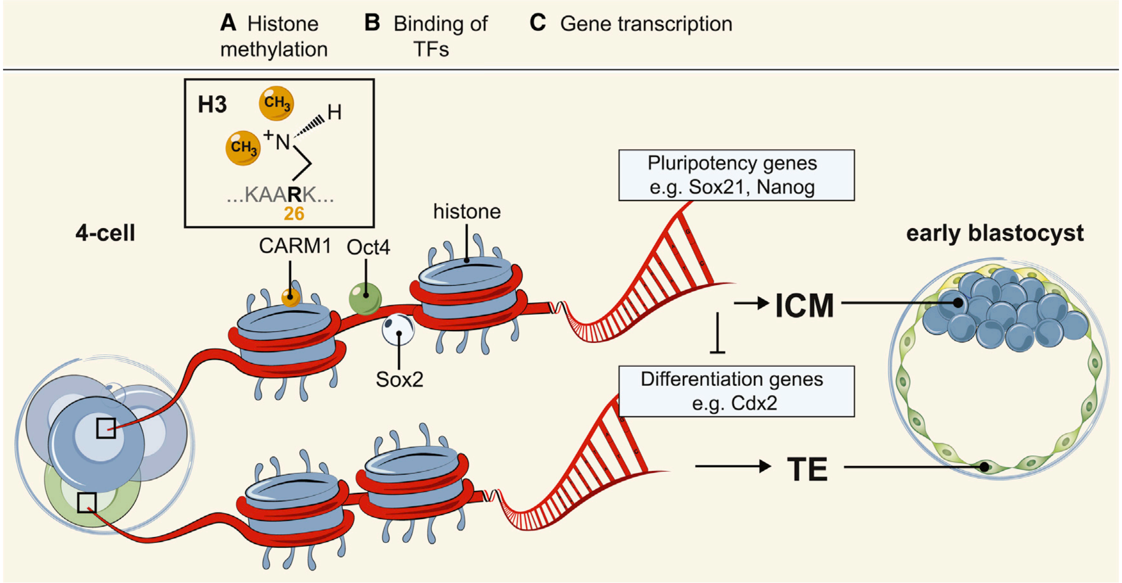

Figure 7. Model of How Cellular Heterogeneities at the 4-Cell Stage Regulate Cell Fate

(A–C) In 4-cell embryos, CARM1, which methylates histone H3R26, is differentially expressed (Torres-Padilla et al., 2007). We hypothesize that higher levels of histone H3R26me facilitate the binding to DNA of pluripotency regulators such as Oct4 and Sox2, resulting in increased transcription of pluripotency-related target genes, such as *Sox21*, *Nanog*, and *Esrrb*, biasing these cells to contribute to the pluripotent lineage. Conversely, in cells with lower levels of histone H3R26me, pluripotency regulators are only able to bind to DNA for shorter periods of time, and therefore their target genes are not as highly expressed. These cells have lower levels of pluripotency and are thus more likely to initiate expression of differentiation genes, such as *Cdx2*, and initiate development into the extra-embryonic TE.

Analysis of Inter- and Intra-embryonic Heterogeneity

To identify genes that show a significantly high variability across blastomeres in the same embryo, we first regressed out the embryo effect by fitting the linear model:

$$g_{ijk} = A_i + B_{ik} + \varepsilon_{ijk}$$

where g_{ijk} is the log-expression value of the gene i in cell j and embryo k, A_i is the average log-expression of gene i across all cells, B_{ik} represents the embryo effect and ε_{ijk} is the fit residual. The expression level of the gene with the embryo effect removed, $\hat{g}_{ijk}$, is equal to $g_{ijk} - B_{ik}$. We then applied the method described above to the $\hat{g}_{ijk}$ to identify highly variable genes.

The intra- and inter-embryonic transcriptional differences were quantified and compared between blastomeres by computing Spearman's correlation coefficient between all pairs of blastomeres within the same embryo and in different embryos at 2-, 4-, and 8-cell stage. Genes with a mean normalized count less than 10 were removed. Only embryos collected in the same batch were used for the calculation of inter-embryonic correlations (Table S1 for a list of samples).

Testing for Enrichment of Oct4 and Sox2 Targets among Highly Variable Genes

We downloaded a list of Oct4 target genes identified in three different publications (Loh et al., 2006; Matoba et al., 2006; Sharov et al., 2008) and selected only those that were targeted by Oct4 in at least two out of three publications. After removing all genes that were never expressed in our data, we tested the significance of the association between the Oct4 target genes and highly variable genes in 2-, 4-, and 8-cell embryos by Fisher's exact test (p values are, respectively, 0.005, 10^{-9} and 4×10^{-6}). Analogously, we tested the enrichment of Sox2 target genes (Sharov et al., 2008) among the highly variable genes and found statistical significance at the 2- and 4-cell stages (p values 0.004 and 10^{-5}), but not at the 8-cell stage (p value 0.26). Finally, the enrichment test run on genes targeted by Oct4 and/or Sox2 gave the following p values for embryos at 2-, 4-, and 8-cell stage, respectively: 2×10^{-4}, 3×10^{-10}, and 3×10^{-5}.

We focused on genes that are highly variable at the 4-cell stage and are targeted by Oct4 and/or Sox2 (including *Sox21* [Chakravarthy et al., 2011; Göke et al., 2011]) and searched for subsets of genes whose expression patterns are coordinated. To this aim, the matrix of pairwise Spearman correlation coefficients was computed and hierarchical clustering was carried out (R function "hclust," default options). The Dynamic Tree Cut algorithm was used to identify clusters of genes (Langfelder et al., 2008) with the R function "cutreeDynamic" in the "dynamicTreeCut" package (default parameters).

Functional Assays

To downregulate Sox21, three *Sox21*-specific siRNAs (QIAGEN) at a total (combined or individual) concentration of 12 μM were injected together with *GFP*, *Gap43-GFP*, or *Gap43-RFP* mRNA (400 ng/ml). AllStars Negative Control siRNA (QIAGEN) was used as a control. The sequences of the siRNAs used are as follows: Sox21 siRNA 1: 5′-CCCGGTTTGTATGTACATAGA-3′, Sox21 siRNA 2: 5′-TACTCTGATTGTACTGTTGAA-3′, Sox21 siRNA 3: 5′-TTGTATGTACATAGATGTATA-3′. To inhibit CARM1 activity embryos were treated from the 2- to 4-cell stage with a chemical inhibitor against CARM1 (Millipore, catalog no. 217531). CARM1 inhibitor was dissolved in DMSO (final concentration of DMSO was 0.005%) and used at a concentration of 9 μM in KSOM. Control embryos were incubated in the equivalent DMSO concentration but in the absence of the inhibitor. To downregulate CARM1 expression, CARM1 stealth siRNAs (Life Technologies - AM16708) were injected at a concentration of 200 nM together with *Gap43-RFP* mRNA (200 ng/ml). CARM1 mRNA was prepared and microinjected as previously described (Torres-Padilla et al., 2007) at a concentration of 500 ng/μl. Embryos were cultured in KSOM under paraffin oil at 37.5°C in a 5% CO_2.

Immunofluorescence

Primary antibodies used were goat anti-Sox17 (R&D Systems), rabbit anti-Nanog (Abcam), mouse anti-Cdx2 (Biogenex), rabbit anti-Histone H3 (Abcam),

rabbit anti-Sox2 (Cell Signaling Technology), rabbit anti-Sox21 (R&D Systems), and rabbit anti-Histone H3 (symmetric di methyl R26; Abcam). ME embryos were used for Sox21 protein quantification at the 4- and 8-cell stages. Immunofluorescence was carried out as described previously (Jedrusik et al., 2008). Multichannel images were acquired using a Leica SP5 confocal microscope, and confocal z stacks were exported to IMAGEJ for image processing, intensity measurements and cell counting. Quantification of fluorescence intensity was done by normalizing to Histone H3 staining and layer-normalizing using the built-in ImageJ function. Intensity was measured in the normalized sections using the ImageJ measure function.

Live Imaging

Following injection with siRNA, embryos were observed on an inverted epifluorescent Zeiss Axiovert 200M microscope with a 20×/0.75 NA objective. Two-channel (green fluorescence and transmitted light) multisection images were acquired every 30 min with a Hamamatsu ORCA ER CCD camera on 16 focal planes every 6 μm, with an exposure of 10 ms for transmitted light and 50 ms for fluorescence. Simi Biocell software was used for cell tracking, as previously described (Bischoff et al., 2008). Cell identification and lineage rendering was done with MovIT software (from the BioEmergences platform).

qRT-PCR

Total RNA was extracted from 8-cell stage embryos using the Arcturus PicoPure RNA isolation kit (Arcturus Bioscience). qRT-PCR was carried out using SYBR Green in a StepOne Plus Real-time PCR machine (Applied Biosystems). Gapdh was used as an endogenous control. Relative mRNA concentrations were calculated using the ddCT method. The following primers were used: Gapdh forward, 5′-AGAGACGGCCGCATCTTC-3′ and reverse, 5′-CCCAATACGGCCAAATCCGT-3′; Cdx2 forward, 5′-AAACCTGTGCGAG TGGATG-3′ and reverse, 5′-TCTGTGTACACCACCCGGTA-3′; Sox21 forward, 5′-GCCGGTGACTCGTGTCTTTA-3′ and reverse, 5′-GAACGGCGGTCATCT CTCAT-3′; Nanog forward, 5′-GGTTGAAGACTAGCAATGGTCTGA-3′ and reverse, 5′-TGCAATGGATGCTGGGATACT −3′; Oct3/4 forward, 5′-TTGGGC TAGAGAAGGATGTGGTT-3′ and reverse, 5′-GGAAAAGGGACTGAGTAGAGT GTGG-3′; Sox2 forward, 5′-CCATCCACCCTTATGTATCCAAG-3′ and reverse, 5′-CGAAGGAAGTGGGTAAACAGCAC-3′.

ACCESSION NUMBERS

The accession number for the sequencing data reported in this paper is ArrayExpress: E-MTAB-3321.

SUPPLEMENTAL INFORMATION

Supplemental Information includes six figures, two tables, and one data file and can be found with this article online at http://dx.doi.org/10.1016/j.cell.2016.01.047.

AUTHOR CONTRIBUTIONS

M.G. carried out the majority of the experiments with help from S.J.L.G. and A.H. S.J.L.G., M.G., and A.J. scored cell divisions and collected individual cells. A.S. analyzed the single-cell transcriptomics data and performed all downstream analyses using these data. I.C.M. prepared the cDNA libraries and performed and analyzed the deep sequencing data. T.V. supervised the cDNA library preparation. J.C.M. oversaw the computational analysis and supervised the study. M.Z.-G. conceived the project, designed and oversaw experiments, and supervised the study. All authors contributed to writing the manuscript.

ACKNOWLEDGMENTS

We are grateful to the Wellcome Trust, EMBL, and CRUK for supporting our work and our colleagues: M. Shahbazi, D. Glover, and F. Antonica for feedback and G. Recher for imaging assistance. T.V. also acknowledges KU Leuven SymBioSys (PFV/10/016).

Received: June 9, 2015
Revised: November 10, 2015
Accepted: January 22, 2016
Published: March 24, 2016

REFERENCES

Anders, S., Pyl, P.T., and Huber, W. (2015). HTSeq—a Python framework to work with high-throughput sequencing data. Bioinformatics *31*, 166–169.

Biase, F.H., Cao, X., and Zhong, S. (2014). Cell fate inclination within 2-cell and 4-cell mouse embryos revealed by single-cell RNA sequencing. Genome Res. *24*, 1787–1796.

Bischoff, M., Parfitt, D.E., and Zernicka-Goetz, M. (2008). Formation of the embryonic-abembryonic axis of the mouse blastocyst: relationships between orientation of early cleavage divisions and pattern of symmetric/asymmetric divisions. Development *135*, 953–962.

Brennecke, P., Anders, S., Kim, J.K., Kołodziejczyk, A.A., Zhang, X., Proserpio, V., Baying, B., Benes, V., Teichmann, S.A., Marioni, J.C., and Heisler, M.G. (2013). Accounting for technical noise in single-cell RNA-seq experiments. Nat. Methods *10*, 1093–1095.

Buettner, F., Natarajan, K.N., Casale, F.P., Proserpio, V., Scialdone, A., Theis, F.J., Teichmann, S.A., Marioni, J.C., and Stegle, O. (2015). Computational analysis of cell-to-cell heterogeneity in single-cell RNA-sequencing data reveals hidden subpopulations of cells. Nat. Biotechnol. *33*, 155–160.

Burton, A., Muller, J., Tu, S., Padilla-Longoria, P., Guccione, E., and Torres-Padilla, M.E. (2013). Single-cell profiling of epigenetic modifiers identifies PRDM14 as an inducer of cell fate in the mammalian embryo. Cell Rep. *5*, 687–701.

Chakravarthy, H., Ormsbee, B.D., Mallanna, S.K., and Rizzino, A. (2011). Rapid activation of the bivalent gene Sox21 requires displacement of multiple layers of gene-silencing machinery. FASEB J. *25*, 206–218.

Deng, Q., Ramsköld, D., Reinius, B., and Sandberg, R. (2014). Single-cell RNA-seq reveals dynamic, random monoallelic gene expression in mammalian cells. Science *343*, 193–196.

Fleming, T.P. (1987). A quantitative analysis of cell allocation to trophectoderm and inner cell mass in the mouse blastocyst. Dev. Biol. *119*, 520–531.

Gardner, R.L. (2002). Experimental analysis of second cleavage in the mouse. Hum. Reprod. *17*, 3178–3189.

Göke, J., Jung, M., Behrens, S., Chavez, L., O'Keeffe, S., Timmermann, B., Lehrach, H., Adjaye, J., and Vingron, M. (2011). Combinatorial binding in human and mouse embryonic stem cells identifies conserved enhancers active in early embryonic development. PLoS Comput. Biol. *7*, e1002304.

Guo, G., Huss, M., Tong, G.Q., Wang, C., Li Sun, L., Clarke, N.D., and Robson, P. (2010). Resolution of cell fate decisions revealed by single-cell gene expression analysis from zygote to blastocyst. Dev. Cell *18*, 675–685.

Hillman, N., Sherman, M.I., and Graham, C. (1972). The effect of spatial arrangement on cell determination during mouse development. J. Embryol. Exp. Morphol. *28*, 263–278.

Jedrusik, A., Parfitt, D.E., Guo, G., Skamagki, M., Grabarek, J.B., Johnson, M.H., Robson, P., and Zernicka-Goetz, M. (2008). Role of Cdx2 and cell polarity in cell allocation and specification of trophectoderm and inner cell mass in the mouse embryo. Genes Dev. *22*, 2692–2706.

Johnson, M.H., and Ziomek, C.A. (1981). The foundation of two distinct cell lineages within the mouse morula. Cell *24*, 71–80.

Johnson, W.E., Li, C., and Rabinovic, A. (2007). Adjusting batch effects in microarray expression data using empirical Bayes methods. Biostatistics *8*, 118–127.

Kiso, M., Tanaka, S., Saba, R., Matsuda, S., Shimizu, A., Ohyama, M., Okano, H.J., Shiroishi, T., Okano, H., and Saga, Y. (2009). The disruption of Sox21-mediated hair shaft cuticle differentiation causes cyclic alopecia in mice. Proc. Natl. Acad. Sci. USA *106*, 9292–9297.

Kuzmichev, A.N., Kim, S.K., D'Alessio, A.C., Chenoweth, J.G., Wittko, I.M., Campanati, L., and McKay, R.D. (2012). Sox2 acts through Sox21 to regulate transcription in pluripotent and differentiated cells. Curr. Biol. *22*, 1705–1710.

Langfelder, P., Zhang, B., and Horvath, S. (2008). Defining clusters from a hierarchical cluster tree: the Dynamic Tree Cut package for R. Bioinformatics *24*, 719–720.

Loh, Y.H., Wu, Q., Chew, J.L., Vega, V.B., Zhang, W., Chen, X., Bourque, G., George, J., Leong, B., Liu, J., et al. (2006). The Oct4 and Nanog transcription network regulates pluripotency in mouse embryonic stem cells. Nat. Genet. *38*, 431–440.

Love, M.I., Huber, W., and Anders, S. (2014). Moderated estimation of fold change and dispersion for RNA-seq data with DESeq2. Genome Biol. *15*, 550.

Mallanna, S.K., Ormsbee, B.D., Iacovino, M., Gilmore, J.M., Cox, J.L., Kyba, M., Washburn, M.P., and Rizzino, A. (2010). Proteomic analysis of Sox2-associated proteins during early stages of mouse embryonic stem cell differentiation identifies Sox21 as a novel regulator of stem cell fate. Stem Cells *28*, 1715–1727.

Matoba, R., Niwa, H., Masui, S., Ohtsuka, S., Carter, M.G., Sharov, A.A., and Ko, M.S. (2006). Dissecting Oct3/4-regulated gene networks in embryonic stem cells by expression profiling. PLoS ONE *1*, e26.

McDole, K., Xiong, Y., Iglesias, P.A., and Zheng, Y. (2011). Lineage mapping the pre-implantation mouse embryo by two-photon microscopy, new insights into the segregation of cell fates. Dev. Biol. *355*, 239–249.

Morris, S.A., Teo, R.T., Li, H., Robson, P., Glover, D.M., and Zernicka-Goetz, M. (2010). Origin and formation of the first two distinct cell types of the inner cell mass in the mouse embryo. Proc. Natl. Acad. Sci. USA *107*, 6364–6369.

Morris, S.A., Guo, Y., and Zernicka-Goetz, M. (2012). Developmental plasticity is bound by pluripotency and the Fgf and Wnt signaling pathways. Cell Rep. *2*, 756–765.

Parfitt, D.E., and Zernicka-Goetz, M. (2010). Epigenetic modification affecting expression of cell polarity and cell fate genes to regulate lineage specification in the early mouse embryo. Mol. Biol. Cell *21*, 2649–2660.

Picelli, S., Faridani, O.R., Björklund, A.K., Winberg, G., Sagasser, S., and Sandberg, R. (2014). Full-length RNA-seq from single cells using Smart-seq2. Nat. Protoc. *9*, 171–181.

Piotrowska, K., Wianny, F., Pedersen, R.A., and Zernicka-Goetz, M. (2001). Blastomeres arising from the first cleavage division have distinguishable fates in normal mouse development. Development *128*, 3739–3748.

Piotrowska-Nitsche, K., and Zernicka-Goetz, M. (2005). Spatial arrangement of individual 4-cell stage blastomeres and the order in which they are generated correlate with blastocyst pattern in the mouse embryo. Mech. Dev. *122*, 487–500.

Piotrowska-Nitsche, K., Perea-Gomez, A., Haraguchi, S., and Zernicka-Goetz, M. (2005). Four-cell stage mouse blastomeres have different developmental properties. Development *132*, 479–490.

Plachta, N., Bollenbach, T., Pease, S., Fraser, S.E., and Pantazis, P. (2011). Oct4 kinetics predict cell lineage patterning in the early mammalian embryo. Nat. Cell Biol. *13*, 117–123.

Samarage, C.R., White, M.D., Álvarez, Y.D., Fierro-González, J.C., Henon, Y., Jesudason, E.C., Bissiere, S., Fouras, A., and Plachta, N. (2015). Cortical tension allocates the first inner cells of the mammalian embryo. Dev. Cell *34*, 435–447.

Sharov, A.A., Masui, S., Sharova, L.V., Piao, Y., Aiba, K., Matoba, R., Xin, L., Niwa, H., and Ko, M.S. (2008). Identification of Pou5f1, Sox2, and Nanog downstream target genes with statistical confidence by applying a novel algorithm to time course microarray and genome-wide chromatin immunoprecipitation data. BMC Genomics *9*, 269.

Shi, J., Chen, Q., Li, X., Zheng, X., Zhang, Y., Qiao, J., Tang, F., Tao, Y., Zhou, Q., and Duan, E. (2015). Dynamic transcriptional symmetry-breaking in pre-implantation mammalian embryo development revealed by single-cell RNA-seq. Development *142*, 3468–3477.

Stegle, O., Teichmann, S.A., and Marioni, J.C. (2015). Computational and analytical challenges in single-cell transcriptomics. Nat. Rev. Genet. *16*, 133–145.

Strumpf, D., Mao, C.A., Yamanaka, Y., Ralston, A., Chawengsaksophak, K., Beck, F., and Rossant, J. (2005). Cdx2 is required for correct cell fate specification and differentiation of trophectoderm in the mouse blastocyst. Development *132*, 2093–2102.

Tabansky, I., Lenarcic, A., Draft, R.W., Loulier, K., Keskin, D.B., Rosains, J., Rivera-Feliciano, J., Lichtman, J.W., Livet, J., Stern, J.N., et al. (2013). Developmental bias in cleavage-stage mouse blastomeres. Curr. Biol. *23*, 21–31.

Takaoka, K., and Hamada, H. (2012). Cell fate decisions and axis determination in the early mouse embryo. Development *139*, 3–14.

Tang, F., Barbacioru, C., Nordman, E., Bao, S., Lee, C., Wang, X., Tuch, B.B., Heard, E., Lao, K., and Surani, M.A. (2011). Deterministic and stochastic allele specific gene expression in single mouse blastomeres. PLoS ONE *6*, e21208.

Tarkowski, A.K. (1959). Experiments on the development of isolated blastomers of mouse eggs. Nature *184*, 1286–1287.

Torres-Padilla, M.E., Parfitt, D.E., Kouzarides, T., and Zernicka-Goetz, M. (2007). Histone arginine methylation regulates pluripotency in the early mouse embryo. Nature *445*, 214–218.

Watanabe, T., Biggins, J.S., Tannan, N.B., and Srinivas, S. (2014). Limited predictive value of blastomere angle of division in trophectoderm and inner cell mass specification. Development *141*, 2279–2288.

Wu, Q., Bruce, A.W., Jedrusik, A., Ellis, P.D., Andrews, R.M., Langford, C.F., Glover, D.M., and Zernicka-Goetz, M. (2009). CARM1 is required in embryonic stem cells to maintain pluripotency and resist differentiation. Stem Cells *27*, 2637–2645.

Xue, Z., Huang, K., Cai, C., Cai, L., Jiang, C.Y., Feng, Y., Liu, Z., Zeng, Q., Cheng, L., Sun, Y.E., et al. (2013). Genetic programs in human and mouse early embryos revealed by single-cell RNA sequencing. Nature *500*, 593–597.

Yamanaka, Y., Lanner, F., and Rossant, J. (2010). FGF signal-dependent segregation of primitive endoderm and epiblast in the mouse blastocyst. Development *137*, 715–724.

Zernicka-Goetz, M., Morris, S.A., and Bruce, A.W. (2009). Making a firm decision: multifaceted regulation of cell fate in the early mouse embryo. Nat. Rev. Genet. *10*, 467–477.

Cell

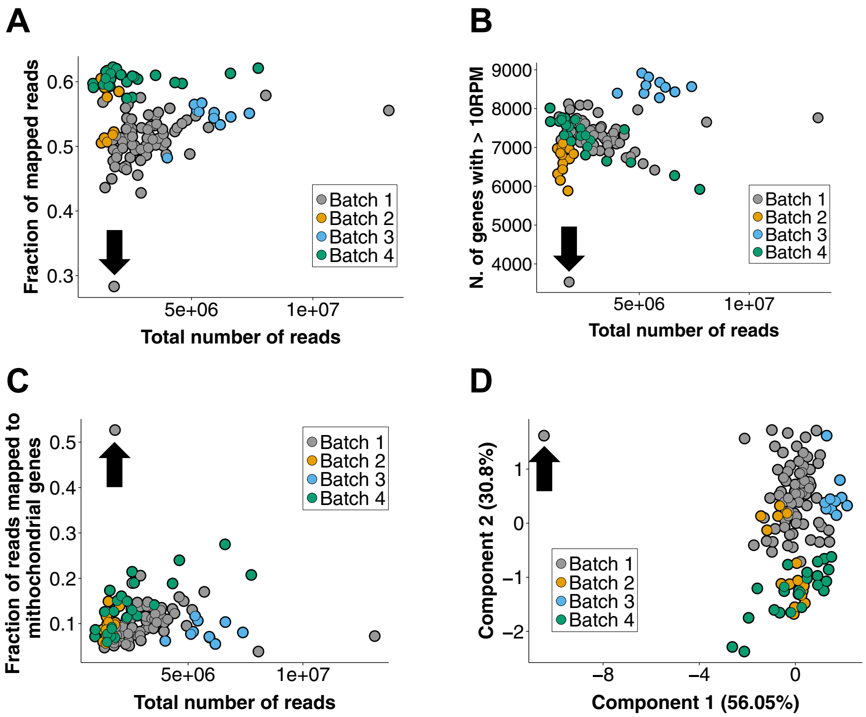

Figure S1. Quality Control Analyses of Single-Cell Transcriptomes, Related to Figure 1

(A–C) The fraction of mapped reads (panel A), the number of genes with more than 10 Reads per Million (panel B) and the fraction of mapped reads allocated to mitochondrial genes were plotted as a function of the total number of reads for all samples. Different colors mark different batches.

(D) Principal Component Analysis of the three metrics plotted in panels A-C. The percentage of variance explained by each principal component is indicated in parentheses. The black arrow in all four panels marks the outlier (sample name “32cell_F”) that was removed from all downstream analysis.

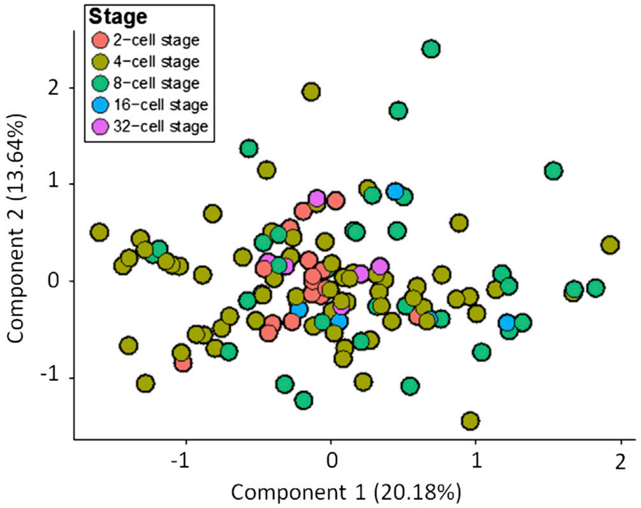

Figure S2. Principal Component Analysis of ERCC Spike-ins from the Samples Shown in Figure 1B, Related to Figure 1

Principal Component Analysis of the log-transformed counts of ERCC spike-ins added to each cells. The numbers in parentheses indicate the percentage of total variance explained by each principal component.

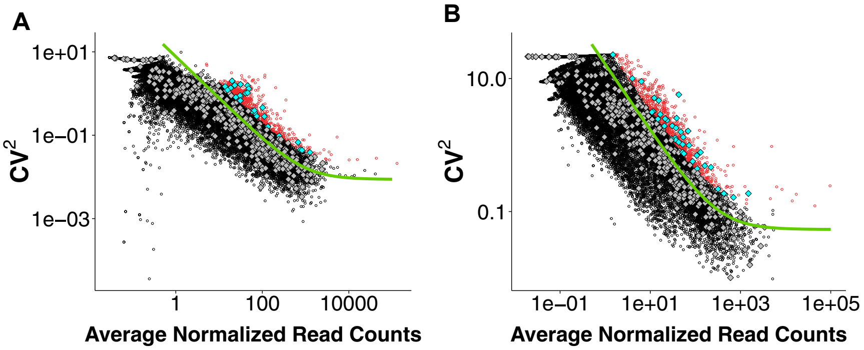

Figure S3. Highly Variable Genes in Embryos at 2- and 8-Cell Stage, Related to Figure 1

Genes displaying a high variability in embryos at 2- (panel A) and 8-cell (panel B) stage were identified, as described in Experimental Procedures. All highly variable genes (with an adjusted p-value < 0.1) are marked by red circles or cyan diamonds. Diamonds mark Oct4/Sox2 target genes.

Cell

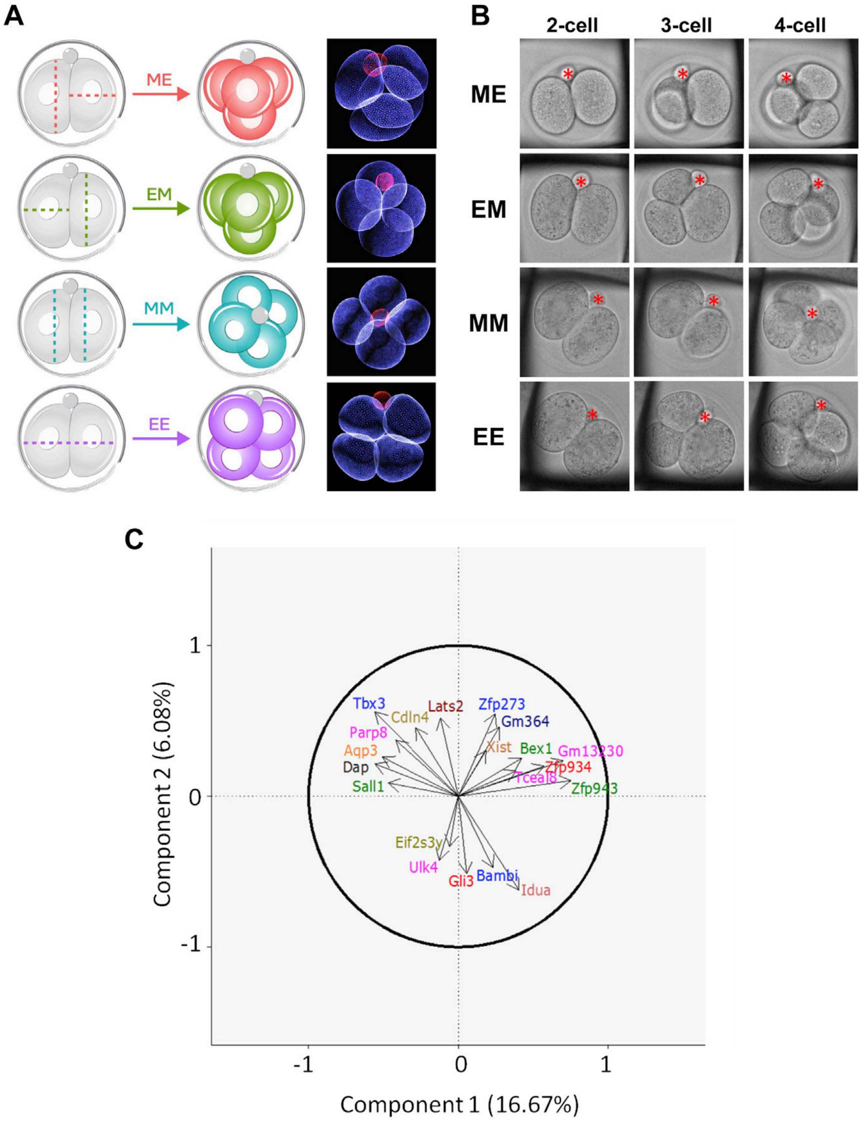

Figure S4. Gene Expression Differences of 4-Cell Stage Embryos with Different Division Patterns, Related to Figure 1

(A) The four different 4-cell stage embryos defined by division pattern.

(B) Live-imaging of embryos to score 2-to 4-cell division pattern. C) 20 genes with the most positive and negative loadings on the first 2 principal components shown in Figure 1C are plotted, with the arrow indicating the correlation with each principal component.

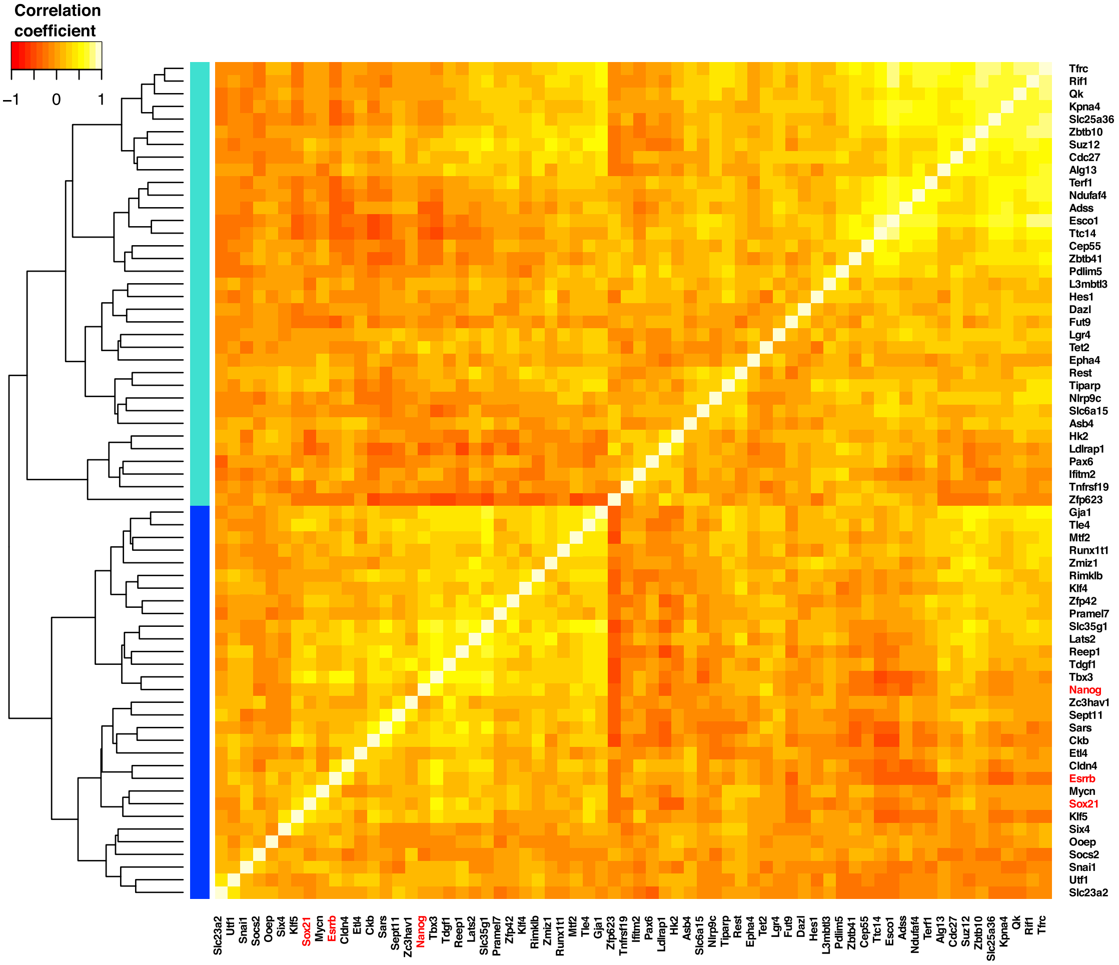

Figure S5. Heatmap Showing the Spearman Correlation Coefficient among Highly Variable Oct4 and/or Sox2 Targets, Related to Figure 2

A dynamic tree cut algorithm identified two clusters of genes showing similar patterns of correlations (colored side bars; Experimental Procedures).

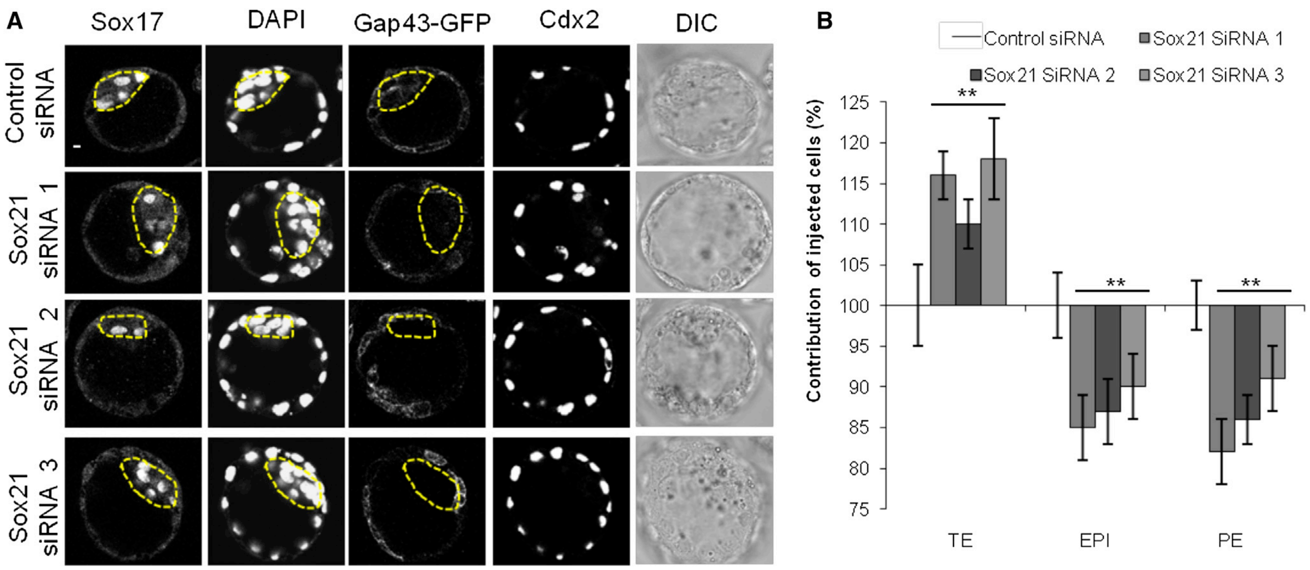

Figure S6. Sox21 Depletion with Three Individual siRNAs Biases Cells toward an Extra-Embryonic Cell Fate, Related to Figure 4
(A) Confocal images of control (n = 24) and Sox21 siRNA 1 (n = 12), Sox21 siRNA 2 (n = 14) and Sox21 siRNA 3 (n = 18) siRNA embryos. Sox17 (PE) and Cdx2 (TE) were used as cell lineage markers. Dotted lines mark the ICM.
(B) Contribution of Sox21 siRNA 1,2 and 3 injected cells to TE, PE and EPI, relative to control siRNA cells.

Cell, Volume *165*

Supplemental Information

Heterogeneity in Oct4 and Sox2 Targets

Biases Cell Fate in 4-Cell Mouse Embryos

Mubeen Goolam, Antonio Scialdone, Sarah J.L. Graham, Iain C. Macaulay, Agnieszka Jedrusik, Anna Hupalowska, Thierry Voet, John C. Marioni, and Magdalena Zernicka-Goetz

Supplemental Tables:

Table S1. List of mRNA sequencing samples, related to Figure 1.

Sample Name	Stage	Division Pattern	Batch	QC
2cell_1_A, 2cell_1_B	2-cell		1	Passed
2cell_2_A, 2cell_2_B	2-cell		1	Passed
2cell_3_A, 2cell_3_B	2-cell		1	Passed
2cell_4_A, 2cell_4_B	2-cell		3	Passed
2cell_5_A, 2cell_5_B	2-cell		3	Passed
2cell_6_A, 2cell_6_B	2-cell		3	Passed
2cell_7_A, 2cell_7_B	2-cell		3	Passed
2cell_8_A, 2cell_8_B	2-cell		3	Passed
ME_4cell_1_A, ME_4cell_1_B, ME_4cell_1_C, ME_4cell_1_D	4-cell	ME	1	Passed
ME_4cell_2_A, ME_4cell_2_B, ME_4cell_2_C, ME_4cell_2_D	4-cell	ME	1	Passed
ME_4cell_3_A, ME_4cell_3_B, ME_4cell_3_C, ME_4cell_3_D	4-cell	ME	1	Passed
ME_4cell_4_A, ME_4cell_4_B, ME_4cell_4_C, ME_4cell_4_D	4-cell	ME	4	Passed
ME_4cell_5_A, ME_4cell_5_B, ME_4cell_5_C, ME_4cell_5_D	4-cell	ME	4	Passed
ME_4cell_6_A, ME_4cell_6_B, ME_4cell_6_C, ME_4cell_6_D	4-cell	ME	4	Passed
EM_4cell_1_A, EM_4cell_1_B, EM_4cell_1_C, EM_4cell_1_D	4-cell	EM	1	Passed
EM_4cell_2_A, EM_4cell_2_B, EM_4cell_2_C, EM_4cell_2_D	4-cell	EM	1	Passed
EM_4cell_3_A, EM_4cell_3_B, EM_4cell_3_C, EM_4cell_3_D	4-cell	EM	1	Passed
EM_4cell_4_A, EM_4cell_4_B, EM_4cell_4_C, EM_4cell_4_D	4-cell	EM	4	Passed
EM_4cell_5_A, EM_4cell_5_B, EM_4cell_5_C, EM_4cell_5_D	4-cell	EM	4	Passed
EM_4cell_6_A, EM_4cell_6_B, EM_4cell_6_C, EM_4cell_6_D	4-cell	EM	4	Passed
MM_4cell_1_A, MM_4cell_1_B, MM_4cell_1_C, MM_4cell_1_D	4-cell	MM	1	Passed

Sample Name	Stage	Division Pattern	Batch	QC
MM_4cell_2_A, MM_4cell_2_B, MM_4cell_2_C, MM_4cell_2_D	4-cell	MM	1	Passed
MM_4cell_3_A, MM_4cell_3_B, MM_4cell_3_C, MM_4cell_3_D	4-cell	MM	1	Passed
EE_4cell_1_A, EE_4cell_1_B, EE_4cell_1_C, EE_4cell_1_D	4-cell	EE	1	Passed
8cell_1_A, 8cell_1_B, 8cell_1_C, 8cell_1_D, 8cell_1_E, 8cell_1_F, 8cell_1_G, 8cell_1_H	8-cell		1	Passed
8cell_2_A, 8cell_2_B, 8cell_2_C, 8cell_2_D, 8cell_2_E, 8cell_2_F, 8cell_2_G, 8cell_2_H	8-cell		1	Passed
8cell_3_A, 8cell_3_B, 8cell_3_C, 8cell_3_D, 8cell_3_E, 8cell_3_F, 8cell_3_G, 8cell_3_H	8-cell		2	Passed
8cell_4_A, 8cell_4_B, 8cell_4_C, 8cell_4_D, 8cell_4_E, 8cell_4_F, 8cell_4_G, 8cell_4_H	8-cell		2	Passed
16cell_A	16-cell		1	Passed
16cell_B	16-cell		1	Passed
16cell_C	16-cell		1	Passed
16cell_D	16-cell		1	Passed
16cell_E	16-cell		1	Passed
16cell_F	16-cell		1	Passed
32cell_A	32-cell		1	Passed
32cell_B	32-cell		1	Passed
32cell_C	32-cell		1	Passed
32cell_D	32-cell		1	Passed
32cell_E	32-cell		1	Passed
32cell_F	32-cell		1	Failed

Table S2. List of genes that are heterogeneous at 2, 4 and 8 cell stages, related to Figure 1.

Ensembl Gene ID	Gene Name
ENSMUSG00000018733	Pex12
ENSMUSG00000024922	Ovol1
ENSMUSG00000035202	Lars2
ENSMUSG00000090744	Gm6871
ENSMUSG00000027079	Clp1
ENSMUSG00000036078	Sigmar1
ENSMUSG00000026049	Tex30
ENSMUSG00000041287	Sox15
ENSMUSG00000001270	Ckb
ENSMUSG00000043421	Hilpda
ENSMUSG00000033948	Zswim5
ENSMUSG00000004359	Spic
ENSMUSG00000021712	Trim23
ENSMUSG00000086324	Gm15564
ENSMUSG00000074733	5830428H23Rik
ENSMUSG00000061371	Zfp873
ENSMUSG00000067813	Xkr9
ENSMUSG00000025921	Rdh10
ENSMUSG00000101655	NA
ENSMUSG00000038496	Slc19a3
ENSMUSG00000028109	Hormad1
ENSMUSG00000064194	Zfp936
ENSMUSG00000101609	NA
ENSMUSG00000041961	Znrf3
ENSMUSG00000001525	Tubb5
ENSMUSG00000001555	Fkbp10
ENSMUSG00000064370	mt-Cytb
ENSMUSG00000051176	Zfp42
ENSMUSG00000033419	Snap91
ENSMUSG00000001558	Klhl10
ENSMUSG00000071302	2610044O15Rik8
ENSMUSG00000017550	Atad5
ENSMUSG00000037904	Ankrd9
ENSMUSG00000064351	mt-Co1
ENSMUSG00000040013	Fkbp6
ENSMUSG00000025838	Pramel6
ENSMUSG00000064337	mt-Rnr1
ENSMUSG00000025068	Gsto1
ENSMUSG00000017548	Suz12
ENSMUSG00000026675	Hsd17b7
ENSMUSG00000012640	Zfp715
ENSMUSG00000025764	Phf17

ENSMUSG00000030747	Dgat2
ENSMUSG00000029569	Tmem168
ENSMUSG00000027954	Efna1
ENSMUSG00000060935	AI597468
ENSMUSG00000019818	Cd164
